# Cellular and subcellular localization of Rab10 and phospho-T73 Rab10 in the mouse and human brain

**DOI:** 10.1186/s40478-023-01704-9

**Published:** 2023-12-18

**Authors:** Vijay Singh, Marissa A. Menard, Geidy E. Serrano, Thomas G. Beach, Hien T. Zhao, Alexis Riley-DiPaolo, Nitya Subrahmanian, Matthew J. LaVoie, Laura A. Volpicelli-Daley

**Affiliations:** 1 Center for Neurodegeneration and Experimental Therapeutics, University of Alabama at Birmingham, Birmingham, AL 35294 USA; 2 Department of Neuropathology, Banner Sun Health Research Institute, Sun City, AZ 85351 USA; 3 Ionis Pharmaceuticals Inc, Carlsbad, CA 92010 USA; 4 Department of Neuroscience at the University of Florida, Gainesville, FL 32611 USA; 5 Department of Neurology, Center for Translational Research in Neurodegenerative Disease, Fixel Institute for Neurologic Disease, University of Florida, Gainesville, FL 32610 USA

**Keywords:** Rab10, pRab10, Antisense oligonucleotide, Rab10 knock down, Phosphorylation, Mouse brain, Parkinson’s disease

## Abstract

Autosomal dominant pathogenic mutations in Leucine-rich repeat kinase 2 (LRRK2) cause Parkinson’s disease (PD). The most common mutation, G2019S-LRRK2, increases the kinase activity of LRRK2 causing hyper-phosphorylation of its substrates. One of these substrates, Rab10, is phosphorylated at a conserved Thr73 residue (pRab10), and is one of the most abundant LRRK2 Rab GTPases expressed in various tissues. The involvement of Rab10 in neurodegenerative disease, including both PD and Alzheimer’s disease makes pinpointing the cellular and subcellular localization of Rab10 and pRab10 in the brain an important step in understanding its functional role, and how post-translational modifications could impact function. To establish the specificity of antibodies to the phosphorylated form of Rab10 (pRab10), Rab10 specific antisense oligonucleotides were intraventricularly injected into the brains of mice. Further, Rab10 knock out induced neurons, differentiated from human induced pluripotent stem cells were used to test the pRab10 antibody specificity. To amplify the weak immunofluorescence signal of pRab10, tyramide signal amplification was utilized. Rab10 and pRab10 were expressed in the cortex, striatum and the substantia nigra pars compacta. Immunofluorescence for pRab10 was increased in G2019S-LRRK2 knockin mice. Neurons, astrocytes, microglia and oligodendrocytes all showed Rab10 and pRab10 expression. While Rab10 colocalized with endoplasmic reticulum, lysosome and trans-Golgi network markers, pRab10 did not localize to these organelles. However, pRab10, did overlap with markers of the presynaptic terminal in both mouse and human cortex, including α-synuclein. Results from this study suggest Rab10 and pRab10 are expressed in all brain areas and cell types tested in this study, but pRab10 is enriched at the presynaptic terminal. As Rab10 is a LRRK2 kinase substrate, increased kinase activity of G2019S-LRRK2 in PD may affect Rab10 mediated membrane trafficking at the presynaptic terminal in neurons in disease.

**Supplementary Information:**

The online version contains supplementary material available at 10.1186/s40478-023-01704-9.

## Introduction

Parkinson’s disease (PD) is the most common neurodegenerative motor disease characterized by Lewy bodies and loss of dopaminergic neurons in the substantia nigra pars compacta (SNc) [4, 52, 76]. Mutations in the LRRK2 gene are the most common genetic cause of PD. The G2019S-LRRK2 mutations accounts for 30–41% of all familial cases, and 10% of sporadic PD [23, 31, 38, 39, 57, 58, 68, 99]. A previous study by our group showed that LRRK2 is expressed in the cortex, SNc, and in the striatum of the rodent brain particularly in layer V pyramidal neurons in the cortex, and spiny projection neurons in the striatum [73, 90]. Most mutations in LRRK2 increase its kinase activity, increasing phosphorylation of a broad subset of Rab GTPases [79]. Rab10 in particular has been validated in multiple studies as a substrate for LRRK2 which phosphorylates threonine 73 (pT73Rab10) in the switch II domain involved in effector binding [78, 79]. Rab10 has been implicated in maintaining ER morphology, ciliogenesis, as well as various steps of membrane trafficking pathways such as vesicle targeting from Golgi to plasma membrane, recycling endosomes, and autophagy pathways [17, 18, 29, 40, 42, 67, 70, 87]. Rab10 also plays a critical role in development as evidenced by the lethality of a Rab10 double knock out in mice [45]. In neurons, Rab10 is involved in polarization, dendrite arborization, and anterograde transport of vesicles along the axon. Additionally, Rab10 is involved in recycling of AMPA receptors at the synapse [14, 21, 43, 88, 93]. In astrocytes and cholinergic interneurons, pRab10 inhibits ciliogenesis and disruptions in sonic hedgehog signaling [15, 30].

In recent years, the role of Rab10 has gained more interest in PD as well as Alzheimer’s disease [3, 19]. In Alzheimer’s disease, a variant in the Rab10 genomic sequence confers protection [83]. Levels of Rab10 are higher in the brains of AD patients compared to healthy controls, and reduced expression of Rab10 decreases levels of Aβ42 in animal models of AD [64]. pRab10 localizes to neurofibrillary tangles in the hippocampus of AD, Down syndrome, and Progressive Supranuclear Palsy [64, 94]. In PD, levels of pRab10 are high in human induced pluripotent stem cells (iPSCs), human induced neurons (iNs), and neutrophils [3, 19, 20].

Despite diverse roles in neuronal function and involvement in neurodegenerative diseases, it is not known in detail where Rab10 and pRab10 are localized in the brain, likely because of its relatively low expression levels [41]. To understand the role of Rab10 in PD and in other neurodegenerative diseases, it is important to characterize the localization of Rab10 and pRab10 in relevant brain areas and in different cell types in the brain. In this study we characterized Rab10 and pRab10 localization at the cellular and subcellular level. We found Rab10 and pRab10 localized to most neuron and glial cell types in the cortex, striatum and midbrain. At a subcellular level, Rab10 localized to the ER, Golgi, and late endosomes, while pT73Rab10 did not overlap with markers of these organelles. Rather, pRab10 was enriched in the presynaptic terminal in both mouse and human cortex. Thus, our findings suggest a role for phosphorylation of Rab10 by LRRK2 in presynaptic vesicle traffic.

## Materials and methods

### Animals

All animal protocols were approved by our University’s Institutional Animal Care and Use Committee. Mice were on a 12-h light/dark cycle and had ad libitum access to food and water. C57BL/6J mice (Jackson labs), G2019S-LRRK2 knock in mice (Jackson labs, strain 030961) on a C57BL/6J background and littermate wild type (WT) controls were used in this study [95]. Both male and female mice were used in this study.

### Rab10 ASOs

ASOs were synthesized as previously described [81] as 20-mer gapmer phosphorothioate oligonucleotides with 2′-MOE groups at positions 1–5 and 15–20. The sequence of Rab10 ASO1 is:GToTToCAGGATATGATmCoGoGmCT, the sequence of Rab10 ASO2 is: TmComCGoAAATATGTGGTAoGoTAmC.:Control ASO is: AAoToGomCTTTmCATAAmCTTomComCAmC. The linkages marked “o” represent the normal phosphodiester (PO), and “mC” represent the 5-methlcystosine. The knock down effect of Rab10 ASO 1 and Rab10 ASO 2 on Rab10 and pRab10 expression was comparable in immunoblot experiments using mouse brain samples (Additional file1 a,b,c,d) therefore, performed all the experiments using Rab10 ASO1.

### Injections

At 3–4 months of age, C57BL/6J WT mice received intraventricular injections with control and Rab10 specific ASOs (Rab10 ASO 1 and Rab10 ASO 2). Mice were deeply anesthetized with vaporized isoflurane on a stereotactic frame. Mice were then injected with 10 µL of 30 µg/µL (in sterile PBS, total 300 µg) control and Rab10 ASOs using the coordinates + 0.2 mm AP, + 1.0 mm ML, −2.4 mm DV. Solutions were injected at a constant rate of 0.5 µL/min; once injection was complete, the needle was left in place for 5 min and then slowly withdrawn.

### Antibodies

Rab10 (4262S, Cell Signaling Technology) for immunoblot, Rab10 (ab237703, Abcam) for immunofluorescence, pRab10 for immunoblot (ab230261, Abcam), pRab10 for immunofluorescence (ab241060, Abcam), Hsc70 (ab19136, Abcam), α-Tubulin (SC23948, Santa Cruz Biotechnology), parvalbumin, (NBP2-50036, NovusBio), NeuN (MAB377, Millipore), SATB2 (ab51502 Abcam), calretinin (MAB1568, Millipore), DARPP32 (MAB4230, R&D Systems), ChAT (NBP2-46620, NovusBio), DAT loop (6-8D6 Santa Cruz Biotechnology), tyrosine hydroxylase (TH) (ab76442, Abcam), CD68 (NBP2-33337SS, NovusBio), GFAP (AB5541, Millipore), Olig2 (MABN50 Millipore), KDEL receptor (sc-58774, Santa Cruz Biotechnology), TGN46 (MA3-063, ThermoFisher), LAMP1 (1D4B, DHSB), EEA1 (NBP2-36568, NovusBio), α-Synuclein (610786, BD Transduction Lab), Synuclein (ab51252, Abcam), VAMP2 (104 211, Synaptic Systems), Homer1 (160 006 Synaptic Systems), Rab8a (ab188574).

### Primary corticostriatal neuron culture and Rab10 ASO treatment

Primary corticostriatal neuron culture from E17-E18 mouse embryo was performed as described previously [85]. Cells were grown in a six well plate in 37 °C incubator with 5% CO_2_ and 95% humidity. Neurons were treated with Rab10 ASO-1 and control ASO at 3 µM final concentration at DIV1. At DIV 14, cells were scraped using lysis buffer containing 1% Triton X-100 (Tx-100), protease and phosphatase inhibitor in 1 × Tris Buffered Saline (TBS) (20 mM Tris, 150 mM NaCl, pH 7.4) at 4 °C. The TX-100 fraction was used for immunoblots.

### Immunoblotting

Mice were anesthetized using isoflurane and transcardially perfused using 0.9% saline, 10 U/mL heparin, and 0.5% *w/v* sodium nitroprusside. Brains were removed and the cortex, midbrain and striatum were isolated and flash frozen using methyl butane and dry ice and stored at −80 °C. Samples were homogenized using sonication (Fisher dismembrator Model 120, 30% amplitude 2 s on, 2 s off for 15–45 s) in lysis buffer containing 1% TX-100, protease and phosphatase inhibitor in 1 × tris buffered saline (TBS) (20 mM Tris, 150 mM NaCl, pH 7.4) at 4 °C. After lysing, samples were spun for 10 min at 4 °C 1000$\times$*g*. The supernatant was diluted into Laemmli buffer with 5% fresh dithiothreitol added. Samples were resolved on 12% SDS-PAGE and transferred to PVDF membrane (Millipore) at 85 V at 4 °C for 90 min using transfer buffer (Tris 25 mM, glycine 192 mM, 20% methanol, pH 8.3). Most blots were blocked using 5% non-fat dry milk in TBS room temperature. pRab10 blots were blocked using “Every Blot” blocking buffer (BioRad 12010020). After blocking, blots were incubated at 4 °C overnight with primary antibodies in blocking buffer. pRab10 blots were incubated with primary antibodies diluted in signal booster kit solution 1 (Fisher 40-720-71KIT). After incubation, blots were washed three times with TBS/0.1% Tween (TBST) and further incubated for 1 h at room temperature in HRP-conjugated secondary antibody in blocking solution. pRab10 blots were incubated with secondary antibodies diluted in signal booster kit solution 2 (Fisher 40-720-71KIT). Following three rinses with TBST, blots were incubated in enhanced chemiluminescence Western Blotting Substrate (Thermofisher) at room temperature and developed using X-ray film.

### Immunofluorescence in mouse brain

Three to four month old mice were anesthetized with isoflurane and transcardially perfused using 0.9% saline, 10U/mL heparin, and 0.5% w/v sodium nitroprusside followed by cold 4% paraformaldehyde in phosphate buffered saline (PBS). Brains were postfixed in 4% PFA in PBS for 16 h at 4 °C, followed by 30% sucrose in PBS for 24–48 h for cryoprotection. Brains were snap frozen using 2-methyl butane and dry ice to lower the temperature up to −35 °C and after freezing, brains were stored at −80 °C until sectioned coronally at 40 µm thickness on a freezing microtome, then stored at −20 °C in cryoprotectant buffer (50% glycerol, 0.01% sodium azide in TBS). For immunofluorescence, sections were rinsed three times in TBS at room temperature followed by incubation in antigen retrieval solution (10 mM sodium citrate, 0.05% Tween-20, pH 6) at 37 °C for 1 h. Sections were rinsed three times in TBS and blocked using 5% normal goat serum (Equitech-Bio Inc) with 0.1% TX-100 in TBS for 1 h at 4 °C. After blocking, sections were incubated in primary antibody solution in 5% normal goat serum in TBS at 4 °C overnight. After three rinses in TBS, sections were incubated in appropriate Alexa-conjugated secondary antibodies (Invitrogen) diluted in blocking solution for 1 h at room temperature. After secondary antibody incubation, sections were washed three times in TBS and mounted using ProLong Gold.

### Tyramide signal amplification

For tyramide signal amplification (TSA) immunofluorescence, mouse brain sections were rinsed three times in TBS at room temperature and treated with 3% H_2_O_2_ solution (diluted in TBS) for 10 min at room temperature. After three rinses in TBS, sections were incubated in antigen retrieval solution (10 mM sodium citrate, 0.05% Tween-20, pH 6) at 37 °C for 1 h. Sections were rinsed three times in 1X TBS and blocked using 5% normal goat serum (Equitech-Bio Inc) with 0.1% TX-100 in 1X TBS for 1 h at 4 °C. After blocking, sections were incubated in pRab10 primary antibody solution in 5% normal goat serum in TBS at 4 °C overnight. After three rinses in TBS, sections were incubated in appropriate Alexa-conjugated secondary antibodies (Invitrogen) in addition to goat anti-rabbit 1 × HRP polymer antibody solution provided with tyramide kit (B40953, Thermo Fisher), for 1 h at room temperature. Sections were rinsed three times in TBS, and incubated with Alexa Fluor 488 tyramide reagent diluted in 3% H_2_O_2_ solution in TBS buffer for 10 min. The reaction was quenched using stop buffer provided in the tyramide kit for one minute at room temperature and rinsed in TBS. Sections were mounted using ProLong Gold.

### Human brain sections and immunofluorescence

The human brain sections were from the Department of Neuropathology, Banner Sun Health Research Institute. The brain sections were from temporal cortex of three subjects, one male and two females, ages > 69 years old. Neuropathologic analyses confirmed the brains to be free from neuritic plaques, neurofibrillary tangles, and Lewy pathology. For tyramide immunofluorescence staining, human brain sections were rinsed three times in PBS at room temperature and treated with 3% H_2_O_2_ solution (diluted in PBS) for 10 min at room temperature. After three rinses in PBS, sections were incubated in antigen retrieval solution (10 mM sodium citrate, 0.05% Tween-20, pH 6) at 37 °C for 1 h. Sections were rinsed three times in 1X PBS and blocked using 5% normal goat serum (Equitech-Bio Inc) with 0.1% TX-100 in 1X PBS for 2 h at 4 °C. After blocking, sections were incubated in primary antibody solution in 5% normal goat serum in PBS at 4 °C overnight. After three rinses in PBS, sections were incubated in appropriate Alexa-conjugated secondary antibodies (Invitrogen) in addition to goat anti-rabbit 1 × HRP polymer antibody solution provided with tyramide kit (B40953, Thermo Fisher), for 2 h at room temperature. Sections were rinsed three times in PBS and incubated with Alexa Fluor 488 tyramide reagent diluted in 3% H_2_O_2_ solution in PBS buffer for 10 min. The reaction was quenched using stop buffer provided in the tyramide kit for one minute at room temperature and rinsed in PBS. Sections were mounted using ProLong Gold.

### Confocal microscopy and image quantitation

Mouse brain sections were imaged using a Nikon A1 laser scanning confocal microscope. At least three images per brain section were obtained from three brain sections from three independent mice. ImageJ software was used for image processing. Colocalization analysis was performed using JACoP plugin in ImageJ. JACoP plugin was run using manual threshold to visualize the appropriate florescence intensity of the sample. The Mander’s colocalization coefficient (MCC) output from JACoP plugin was plotted using GraphPad Prism 9 software. For presynaptic and postsynaptic distance measurement synapses were identified using a presynaptic marker VAMP2 and postsynaptic marker Homer1 in a juxtaposed position in one plane. For distance measurements, ImageJ software was used to draw a perpendicular line across pre- and post-synapse and plot profile tool was used to generate a distance profile to calculate a relative distance between pre- and post-synaptic marker.

### Rab10 knock out culture and immunofluorescence

Rab10 knockout (KO) and WT human cortical iNs were provided by Dr. Matthew J. LaVoie. These cells were grown and differentiated into neurons with a neurogenin2 (NGN2)-induced differentiation protocol as described previously [33, 69]. In brief, following differentiation, frozen day 4 induced neurons were provided on dry ice by the LaVoie lab. Rab10 KO and WT cells were grown and maintained in 24-well plates with glass coverslips at a density of 100 k cells per well. Cells were grown for 12 days at 37 °C and 5% CO2, with greater than 95% humidity, in a cell culture incubator. Cells were revived and grown in neurobasal media supplemented with (dextrose 0.3%, glutamine 1%, non-essential amino acids 0.5%, B27 supplement 2%, BDNF (10 ng/mL), GDNF (10 ng/mL), CNTF (10 ng/mL), puromycin 10 µg/mL, doxycycline (2 µg/mL), ROCK inhibitor (10 µM)). Next day, exchange neurobasal media with all supplements (added 0.5 µM cytarabine) without ROCK inhibitor. Until day 10, every other day a half media change was performed. Day 11 complete neuronal media change was performed with all supplements without puromycin and doxycycline. At day 13, cells were fixed with 4% PFA for 15 min at room temperature and immunofluorescence was performed. After fixing, cells were washed four times with 0.05% saponin in 1 × PBS and blocked using blocking solution (3% bovine albumin serum + 0.05% saponin in 1 × PBS) for 30 min at room temperature. After blocking, cells were incubated in pRab10 and VAMP2 primary antibodies in blocking solution at 4 °C overnight. Next day, after four rinses in 0.05% saponin/PBS, cells were incubated in anti-mouse Alexa555-conjugated secondary antibodies (Invitrogen) in addition to goat anti-rabbit 1 × HRP polymer antibody solution provided with tyramide kit (B40953,Thermo Fisher), for 1 h at room temperature. Cells were rinsed four times in 0.05% saponin solution and incubated with Alexa Fluor 488 tyramide reagent diluted in 3% H_2_O_2_ solution in TBS buffer for 10 min. The reaction was quenched using stop buffer provided in the tyramide kit for one minute at room temperature and rinsed in 1 × PBS. Cells were mounted using ProLong Gold.

### Statistics

All statistical analyses were performed using Graph-Pad Prism software. The following tests were performed: two-tailed Welch’s t-test, two-tailed Mann Whitney test, two-tailed Nested t-test, or one-way ANOVA using Dunnett’s multiple comparisons test. The data was plotted as mean with standard error of the mean. The significance value of the analyses was indicated in the *p* value. Each statistical analysis test is mentioned in the respective results section and in the figure legend.

## Results

### Validation of Rab10 and pRab10 antibodies in mouse brain

**Fig. 1:**
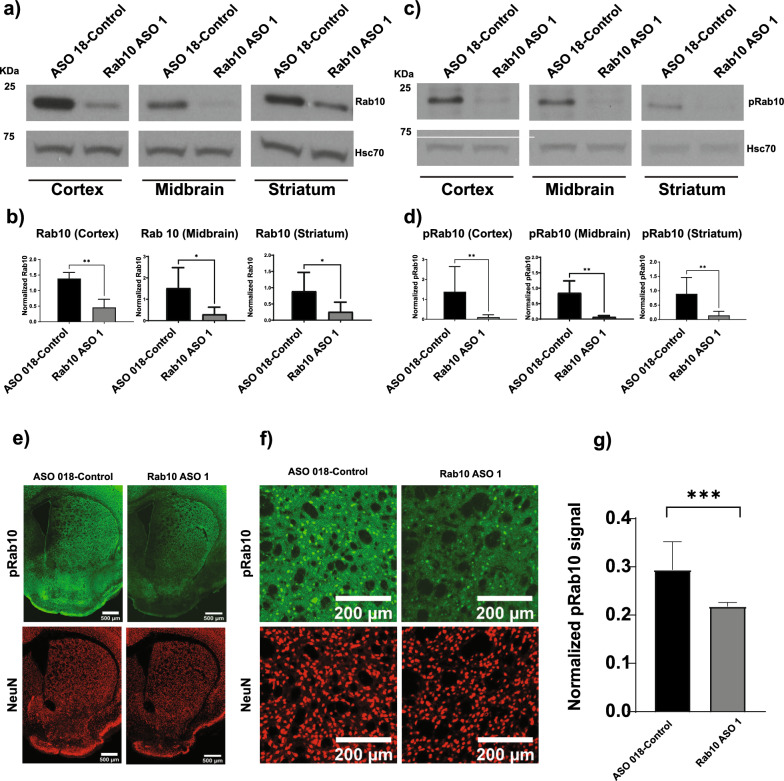
Validation of Rab10 and pRab10 antibodies in the brain using Rab10ASOs C57BL/6JWT mice were intracerebroventricularly injected with control and Rab10 ASO1 to confirm the specificity of the Rab10 and pRab10 antibodies. **a** Immunoblot of Rab10 in Rab10 ASO (Rab10 ASO1) and control ASO injected brain samples for cortex, midbrain and striatum. Hsc70 was used as a loading control. **b** Quantitation of Rab10 protein and normalization with the loading control, Hsc70, showed reduction of Rab10 in brain regions from the Rab10 ASO1 injected mouse (gray bar) compared to control ASO (black bar) in the cortex (*p* value = 0.002), midbrain (*p* value = 0.04) and in the striatum (*p* value = 0.06). Nonparametric Mann–Whitney test was run for statistical analyses (n = 6 biologically independent samples). **c** Immunoblots showed reduction of pRab10 in Rab10 ASO injected brain samples (Rab10 ASO1) compared to control ASO injected in brain samples in the cortex, midbrain and striatum. Hsc70 was used as a loading control. **d** Quantitation of pRab10 protein and normalization with the loading control, Hsc70, showed reduction of pRab10 in the Rab10 ASO1 injected mouse samples (gray bar) compared to control ASO injected mouse samples (black bar) in the cortex (*p* value = 0.004), midbrain (*p* value = 0.002) and in the striatum (*p* value = 0.004). Nonparametric Mann–Whitney test was run for statistical analyses (n = 6 biologically independent samples). **e** Immunofluorescence staining for pRab10 (green) in the striatum showed a reduction of pRab10 in the Rab10 ASO injected mice compared to control ASO injected mice. NeuN (red) immunofluorescence is used to help visualize location on neuronal nuclei and comparable staining in the brain area. Scale bar = 500 µm. All images were captured at the same laser power, gain and offset. **f** The zoom image shows pRab10 (green) reduction in the Rab10 ASO injected sample compared to control ASO sample. Scale bar = 200 µm. **g** Normalization of pRab10 integrated density immunofluorescence signal with NeuN signal and quantitation using nested independent t-test showed a significant reduction (*p* value = 0.0008) in the Rab10 ASO injected samples (gray bar) compared to control samples (black bar) (n = 3 biologically independent samples)

To validate the specificity of Rab10 and pRab10 antibodies, Rab10 and control ASOs were injected intracerebroventricularly into C57BL/6J mice to knockdown levels of Rab10 and pRab10. One-month post-injection, mice were perfused with saline, and the brain was dissected to isolate cortex, midbrain, and striatum regions for immunoblots using antibodies to Rab10 (4262S, CST) and pRab10 (ab230261, Abcam). Immunoblots showed that Rab10 and pRab10 levels were reduced in Rab10 ASO1 injected brain samples compared to control ASO samples (Fig. 1a, c). Quantification of Rab10 immunoblots showed significant reductions of Rab10 in the cortex (*p* value = 0.002), midbrain (*p* value = 0.04), and in the striatum (*p* value = 0.06) (Fig. 1b). Similarly, pRab10 levels were significantly reduced in the cortex (*p* value = 0.004), midbrain (*p* value = 0.002), and in the striatum (*p* value = 0.004) in Rab10 ASO1 injected mice compared to control ASO injected mice (Fig. 1d).

The pRab10 antibody was also tested for immunofluorescence in brain sections. To overcome the weak immunofluorescence signal for pRab10 when using standard AlexaFluor-conjugated antibodies, we utilized the tyramide signal amplification technique to increase specific pRab10 staining. Tyramide reacts with horseradish peroxidase to deposit fluorescent molecules near a target protein. This technique combined with citric acid antigen retrieval resulted in detection of pRab10 signal in brain sections. To confirm the specificity of the pRab10 antibody (ab241060, Abcam), recommended for immunofluorescence, a separate cohort of C57BL/6J mice were injected intracerebroventricularly with Rab10 ASO1 and control ASO for immunofluorescence experiments. Quantitation of pRab10 immunofluorescence signal was performed using ImageJ software. pRab10 fluorescence integrated density was normalized with NeuN integrated density and the pRab10 normalized integrated density was plotted and analyzed using Graph-Pad Prism using two tailed Nested t-test. Data analysis showed a significant reduction (*p* value = 0.0008, N = 9, three brain sections from three mice for each condition of pRab10 in Rab10 ASO1 injected mice compared to control ASO injected mice (Fig. 1e, f, g).

To further confirm the Rab10 ASO specificity, primary corticostriatal neurons were treated with control and Rab10 ASOs as described in the methods section. Immunoblots for Rab8a normalized signal with Hsc70 signal showed that there was no decrease in the Rab8a levels in Rab10 ASO treated samples compared to control ASO samples (Additional file 2 e, f), indicating that the Rab10 ASO did not reduce Rab8a levels. The statistical analysis using unpaired t-test with Welch’s correction shows no significant difference in the Rab8a level in the control ASO and Rab10 ASO treated samples (*p* value = 0.56). These same samples were tested for Rab10 levels using immunoblots and the results showed there was a significant decrease in the Rab10 level in Rab10 ASO treated condition compared to control ASO condition (*p* value = 0.0001, N = 3) (Additional file 2 e, g).

Because the G2019S-LRRK2 mutation increases LRRK2 kinase activity [41, 78, 91], immunofluorescence for pRab10 was performed using G2019S-LRRK2 knockin (KI) mice compared to WT littermates. Immunofluorescence of pRab10 was quantified in the striatum because LRRK2 is highly expressed in this brain region [90]. For these experiments, the integrated density of the pRab10 fluorescence was calculated using ImageJ software and normalized with NeuN. Indeed, the two tailed Nested t-test analysis shows that pRab10 immunofluorescence was significantly higher in G2019S-LRRK2 KI striatum compared to WT controls (*p* value = 0.0042, N = 6, two brain sections from three mice for WT and G2019S-LRRK2 KI) (Additional file 1 e, f, g). Due to prior reports showing that Rab10 knockout (KO) mice are embryonic lethal [45], Rab10 KO induced neurons from human iPSCs were used to confirm the specificity of the pRab10 antibody. These cells were induced to differentiate into neurons as reported [33, 47, 69] and immunofluorescence experiments were performed using the pRab10 antibody. In the WT iNs, pRab10 showed punctate immunofluorescence with some overlap with the presynaptic marker, VAMP2. In the Rab10 KO iNs, although there was some signal, in any given area of the confocal image, the signal in Rab10 KO was far less compared to WT cells (Additional file 2 a, b). To evaluate if the tyramide signal amplification contributed any background signal, immunofluorescence experiments were performed with or without the pRab10 antibody with all other steps held constant. In the experimental condition, the pRab10 primary antibody was used and in the control condition, no pRab10 antibody was added after the blocking step. All other reagents including the HRP-conjugated secondary, tyramide reagent and other secondary antibodies were used. Results from the “no-pRab10” antibody condition show that there is minimal background signal without primary antibody (Additional file 2 d) compared to pRab10 antibody condition (Additional file 2 c). This allows us to interpret the signal that is present as specific to the pRab10 antibody and not an artifact of the amplification (Additional file 2 a, c).

### Rab10and pRab10 localization in cortex

**Fig. 2:**
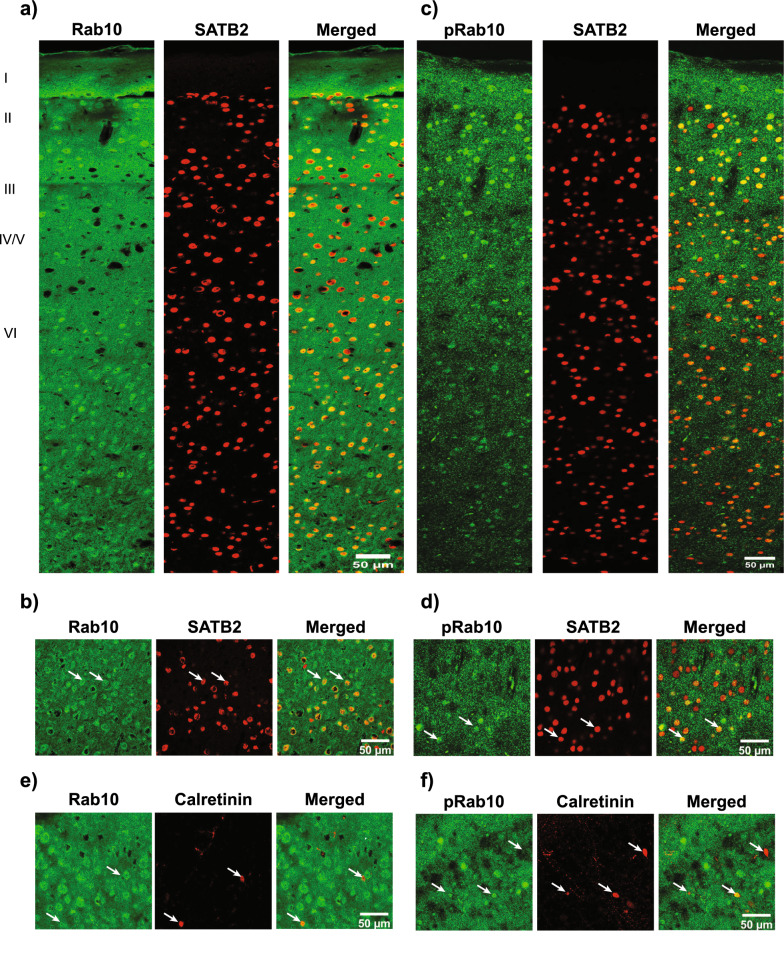
Localization of Rab10 and pRab10 in excitatory neurons and interneurons the cortex. C57BL/6JWT mice at 3–4 months age were used for immunofluorescence localization in the cortex. **a** Rab10 (green) was present in all cortex layers. SATB2, a marker for excitatory neurons, was visualized in red. The merged image showed Rab10 (green) was localized in SATB2 positive excitatory neurons expressing neurons. Scale bar = 50 µm. **b** Rab10 (green) was localized in SATB2 (red) expressing excitatory neurons. Scale bar = 50 µm. **c** pRab10 (green) was present in all cortex layers. SATB2 (red) was expressed in expressed in all cortex layers except layer I and merged image shows pRab10 (green) is localized in SATB2 (red) expressing neurons. Scale bar = 50 µm. **d** High magnification image showed that pRab10 (green) was enriched in SATB2 (red) expressing excitatory neurons. Scale bar = 50 µm. **e** A representative high magnification image showed Rab10 is expressed in calretinin expressing inhibitory neurons indicated by arrows. Scale bar = 50 µm. **f** A representative high magnification image shows pRab10 was expressed in calretinin expressing inhibitory neurons indicated by arrows. Scale bar = 50 µm. (n = 3 biologically independent mouse brain samples)

Immunofluorescence for Rab10 and pRab10 was performed using cortical sections from C57BL/6J WT mice to determine the pattern of expression in the cortex. Rab10 and pRab10 were present in soma and diffuse neuropil in all cortical layers (Fig. 2a, c). Immunofluorescence for special AT-rich sequence binding protein2 (SATB2), a marker of excitatory neurons [27], was present in all layers except for layer 1 which is mostly composed of dendrites and axons (Fig. 2 a, c). Higher magnification images show that Rab10 (Fig. 2b) and pRab10 (Fig. 2d) were enriched in SATB2 expressing excitatory neurons in the cortex (Fig. 2b, d). Rab10 and pRab10 are also present in inhibitory neurons, detected by calretinin as a marker (Fig. 2e, f).

### Rab10 and pRab10 expression in striatum

**Fig. 3:**
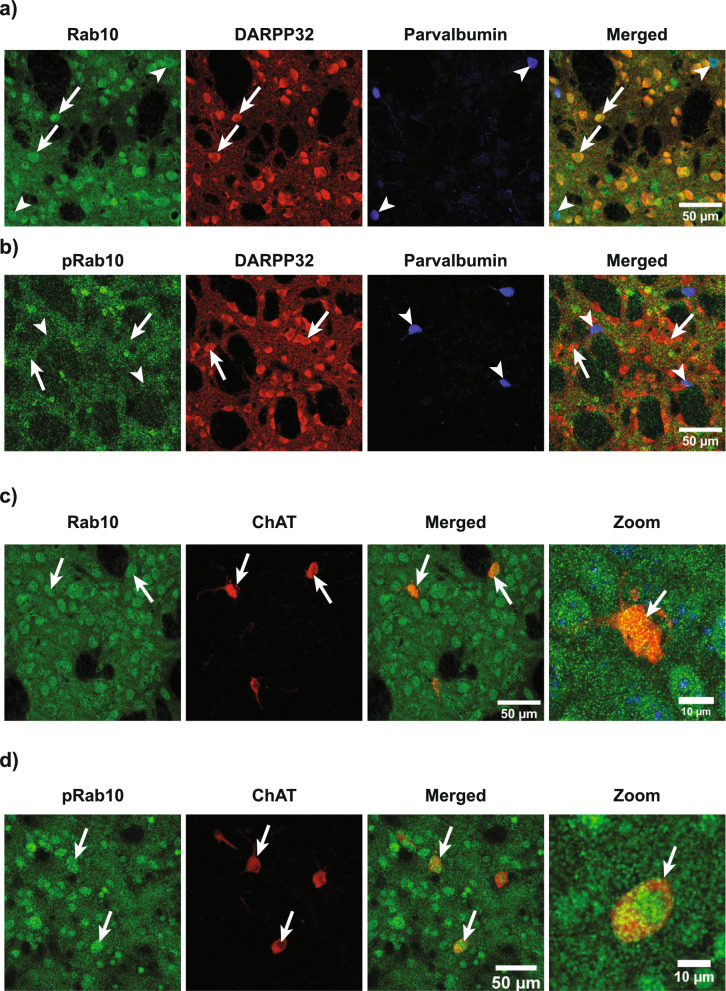
Rab10 and pRab10 immunofluorescence and confocal microscope images of C57BL/6J WT mouse brain striatum. **a** Rab10 (green) expression in DARPP32 (red) positive SPNs indicated by arrows, and parvalbumin (blue) positive interneurons indicated by arrowheads. Scale bar = 50 µm. **b** pRab10 (green) expression in DARPP32 (red) positive SPNs indicated by arrows and parvalbumin (blue) positive interneurons indicated by arrowheads. Scale bar = 50 µm. **c** A representative image shows Rab10 (green) expression in ChAT (red) positive interneurons in merged and higher magnification images indicated by arrows. Scale bar = 50, 10 µm. **d** A representative image shows pRab10 (green) expression in ChAT (red) positive interneurons in merged and higher magnification images indicated by arrows. Scale bar = 50, 10 µm. (n = 3 biologically independent mouse brain samples)

A previous study from our lab showed that LRRK2 is expressed in striatal spiny projection neurons (SPNs) which comprise 95 to 98% of neurons in the striatum [22, 56]. Rab10 is a LRRK2 kinase substrate [79], therefore, immunofluorescence was performed to see if Rab10 and pRab10 are present in SPNs. Rab10 and pRab10 immunofluorescence overlapped with the SPN marker, DARPP32, indicated by arrows (Fig. 3a, b) Other than SPNs, Rab10 and pRab10 were also present in GABAergic interneurons, which make up 2–3% of cells in the striatum [66] Rab10 and pRab10 colocalized with parvalbumin-positive interneurons, in the striatum indicated by arrowhead (Fig. 3a, b). Rab10 and pRab10 immunofluorescence also overlapped with choline acetyltransferase (ChAT), a marker of cholinergic interneurons (Fig. 3c, d), which make up about 1.7% of the cell population in the rat striatum [1, 9, 61, 65, 82].

### Rab10 and pRab10 expression in SNc

**Fig. 4:**
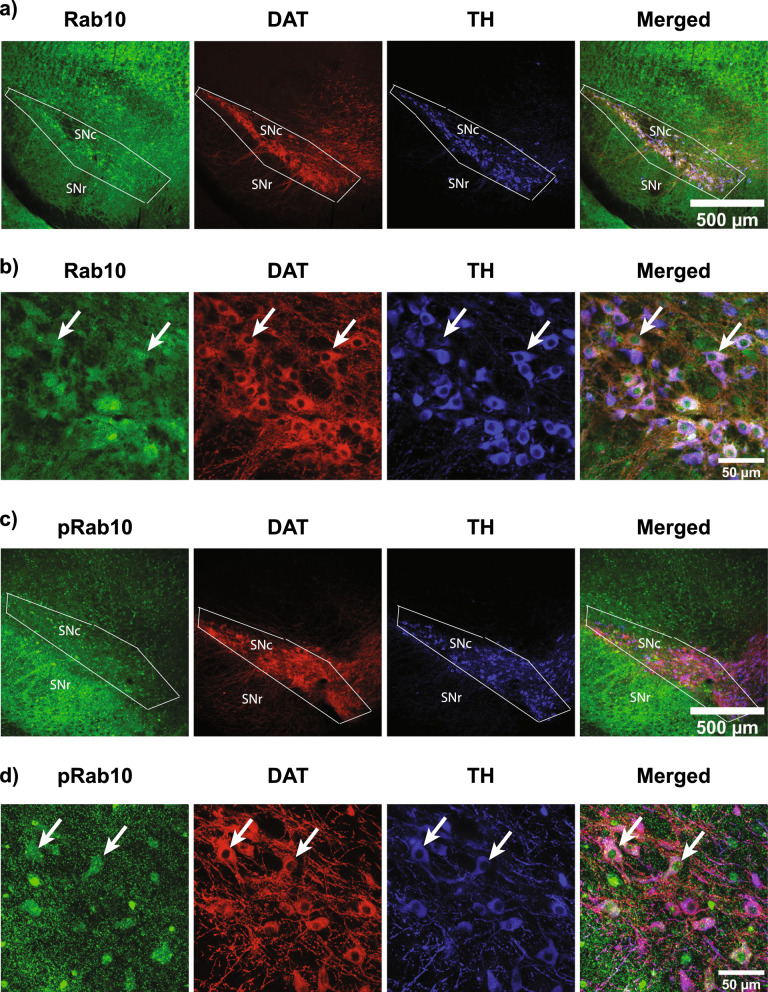
Rab10 and pRab10 immunofluorescence and confocal microscopy images in C57BL/6J WT mouse SNc sections. **a** Low magnification confocal image shows Rab10 (green) is expressed in the SNc brain area, surrounded by a box. DAT (red) and TH (blue) immunofluorescence highlights the SNc brain and merged channel image shows Rab10 expression in this region. Scale bar = 500 µm. **b** Higher magnification image shows Rab10 (green) enrichment in DAT (red) and TH (blue) immuno-positive cells in SNc, indicated by arrows Scale bar = 50 µm. **c** Low magnification confocal image shows pRab10 (green) is expressed in the SNc. DAT (red) and TH (blue) immunofluorescence to highlight the SNc and merged channel image shows pRab10 expression in this region Scale bar = 500 µm. **d** Higher magnification image shows pRab10 (green) enrichment in DAT (red) and TH (blue) immuno-positive cells in the SNc indicated by arrows. Scale bar = 50 µm**.** (n = 3 biologically independent mouse brain samples)

As dopaminergic cell loss in SNc is a hallmark of PD pathology, and mutant LRRK2 causes PD, it is important to determine where LRRK2 substrates, Rab10 and pRab10 localize to the SNc. Immunofluorescence experiments were performed in C57BL/6J WT mouse brain sections. Low magnification images show that both Rab10 and pRab10 were expressed in the SNc (Fig. 4a, c). pRab10 also showed localization to dendrites from the SNc that extend into the substantia nigra pars reticulata (SNr). High magnification images show that Rab10 and pRab10 immunofluorescence overlaps with dopaminergic neuron markers, dopamine transporter (DAT) and tyrosine hydroxylase (TH) immunopositive neurons in the SNc (Fig. 4b, d). Therefore, Rab10 and pRab10 are expressed in SNc dopamine neurons that are susceptible in PD.

### Expression of Rab10 and pRab10 in Glia cells

**Fig. 5:**
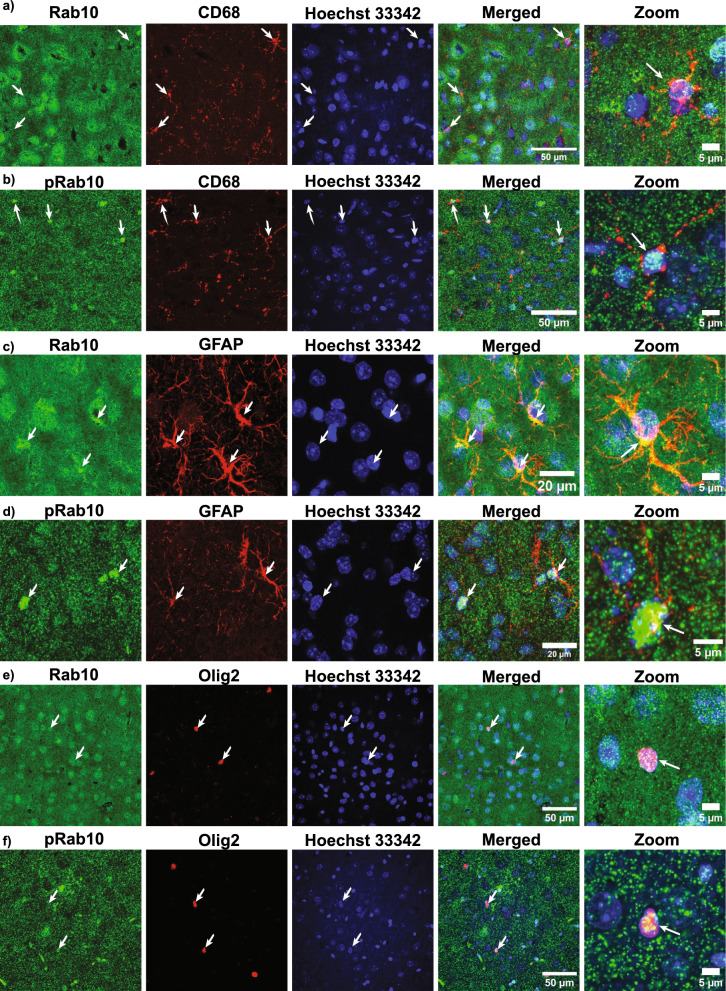
Rab10 and pRab10 immunofluorescence in Glia cell types. Confocal laser scanning microscopy images of C57BL/6J WT mouse brain sections from cortex. **a** A representative confocal image shows Rab10 (green) expression in CD68 (red) positive microglia cells shown in both the merged lower and higher magnification images indicated by arrow. Scale bar = 50, 5 µm. **b** A representative confocal image shows pRab10 (green) expression in CD68 (red) positive cells shown in both the merged lower and higher magnification images indicated by arrow. Hoechst 33,342 staining (blue) shows the cell nucleus. Scale bar = 50, 5 µm. **c** A representative image shows Rab10 (green) expression in GFAP expressing astrocytes (red) shown in the merged lower and higher magnification images indicated by arrows. Hoechst 33,342 staining (blue) shows the cell nucleus. Scale bar = 20, 5 µm. **d**. A representative image shows pRab10 (green) expression in GFAP expressing astrocytes (red) shown in merged lower and higher magnification images indicated by arrows. Hoechst 33,342 staining (blue) shows the cell nucleus. Scale bar = 20, 5 µm. **e** A representative image shows Rab10 (green) expression in Olig2 expressing oligodendrocyte cells (red) shown in merged lower and higher magnification images indicated by arrows. Hoechst 33,342 staining (blue) shows the cell nucleus. Scale bar = 50, 5 µm. **f** A representative image shows pRab10 (green) expression in Olig2 expressing oligodendrocyte cells (red) shown in merged lower and higher magnification images indicated by arrows. Hoechst 33,342 staining (blue) shows the cell nucleus. Scale bar = 50, 5 µm. (n = 3 biologically independent mouse brain samples)

Glial cells make up roughly half of the cells in the central nervous system (CNS) and LRRK2 has been shown to localize to microglia and astrocytes [30, 55, 80, 97]. To examine the expression of the LRRK2 substrate, Rab10, in different glial cell types, immunofluorescence for Rab10 and pRab10 was performed in WT C57BL/6J mouse brain sections from the cortex using different glial cell markers. To examine the expression of Rab10 and pRab10 in microglia, double labeling immunofluorescence was performed using an antibody to CD68, a LAMP (lysosome associated membrane protein) family member, expressed in microglial cells (Fig. 5a, b). Additional file movie 4, 5 showed that Rab10 and pRab10 are expressed in CD68 expressing microglia cells. Immunofluorescence with an antibod.

y specific to GFAP, a marker for astrocytes, revealed expression of Rab10 and pRab10 in astrocytes (Fig. 5c, d). Three dimensional image analyses showed that Rab10 and pRab10 were present in astrocytes (Additional file movie 6, 7). Immunofluorescence with an antibody to Olig2, a marker for oligodendrocytes, showed that Rab10 and pRab10 both are also expressed in oligodendrocytes (Fig. 5e, f). Thus, Rab10 and pRab10 are expressed in all main glia cell subtypes in the brain.

### Rab10 and pRab10 in sub-cellular compartments

**Fig. 6:**
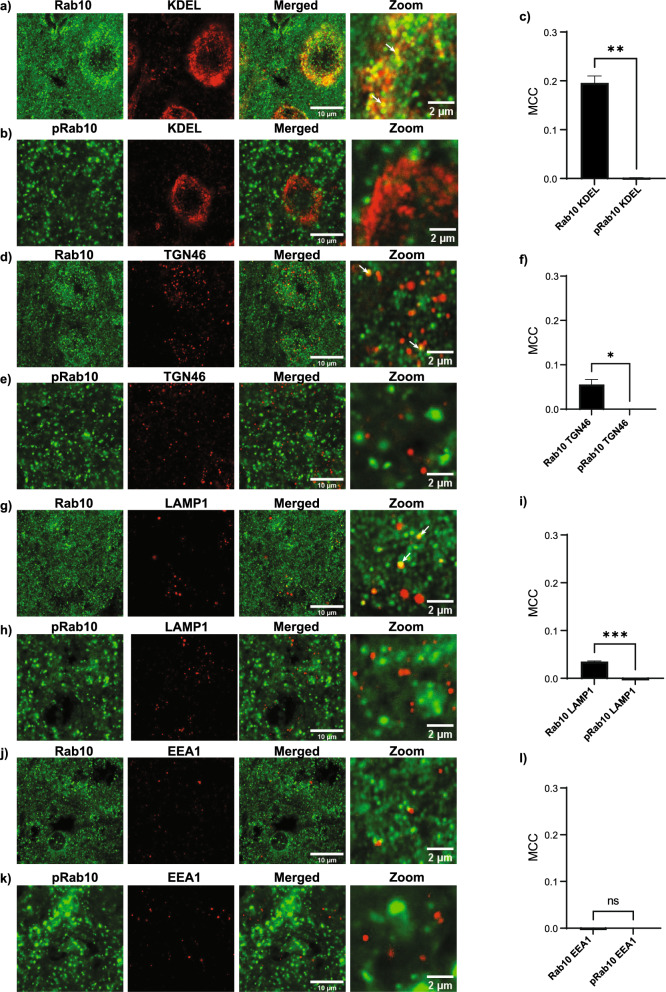
Immunofluorescence confocal images showing Rab10 and pRab10 expression in subcellular compartments in the C57BL/6J WT mouse cortex. **a** Rab10 (green) colocalized with the endoplasmic reticulum (ER) marker, KDEL (red) shown in merged and zoomed in images. Scale bar = 10, 2 µm. **b** pRab10 (green) did not show colocalization with the ER marker, KDEL (red) shown in merged and zoomed in image. Scale bar = 10, 2 µm. **c** Colocalization analyses using Mander’s colocalization coefficient (MCC) indicated a significant difference Rab10 protein colocalization with KDEL compared to pRab10 (*p* value = 0.001). **d** Rab10 (green) colocalized with the trans Golgi network (TGN) marker, TGN46 (red) shown in merged and zoomed in image. Scale bar = 10, 2 µm. **e** pRab10 (green) did not show colocalization with TGN46 (red) shown in merged and zoomed in image. Scale bar = 10, 2 µm. **f** Colocalization analyses using MCC indicated a significant difference in Rab10 protein colocalization with TGN46 compared to pRab10/TGN46 (*p* value = 0.01). **g** Rab10 (green) colocalized with the lysosomal marker, LAMP1 (red) shown in merged and zoomed in image. Scale bar = 10, 2 µm. **h** pRab10 (green) did not show colocalization with LAMP1 shown in merged and zoomed in image. Scale bar = 10, 2 µm. **i** Colocalization analyses using MCC indicated a significant difference in Rab10 protein colocalization with LAMP1 compared to pRab10 (*p* value = 0.004) **j** Rab10 (green) did not show colocalization with the early endosome marker, EEA1 (red) shown in merged and zoomed in image. Scale bar = 10, 2 µm. **k** pRab10 (green) did not show colocalization with EEA1 shown in merged and zoomed in image Scale bar = 10, 2 µm. **l** There was no significant difference between Rab10 and pRab10 colocalization signal with EEA1 indicated by MCC (*p* value = 0.4). Colocalization analysis was performed using ImageJ plug in, JACoP, and unpaired t-test with Welch’s correction was used to get the significance value. (n = 3 biologically independent mouse brain samples)

To understand the involvement of Rab10 and pRab10 in membrane trafficking in neurons, it is important to know their localization to organelles at the subcellular level. Rab10 has been shown to localize at ER membranes and play a role in ER dynamics and maintaining morphology [17]. Here, sections of the cortex were analyzed via immunofluorescence for different organelles using three mouse brains (three sections from each mouse). Immunofluorescence data showed that Rab10 colocalizes with the ER marker, KDEL (Fig. 6a) but its phosphorylated form, pRab10, did not show a significant colocalization with KDEL (Fig. 6b). Mander’s colocalization coefficient (MCC) showed a significant overlap of Rab10 with KDEL (MCC = 0.196) compared to colocalization between pRab10 and KDEL (MCC = 0.0018, p = 0.001) suggesting after phosphorylation, Rab10 does not localize to the ER membrane (Fig. 6c). In addition, previously it has been shown that Rab10 is involved recycling and present at the trans-Golgi network (TGN) [12, 72]. In the present study, Rab10 partially colocalized with TGN46, a TGN marker, (Fig. 6d), but pRab10 did not show a significant colocalization with TGN46 (Fig. 6e). Quantitation of colocalization showed a MCC = 0.056 for Rab10/TGN46, compared to pRab10/TGN46 (MCC = 0.0001, p = 0.01) (Fig. 6f). Furthermore, Rab10 partially colocalized with LAMP1, marker for late endosomes and lysosomes [37, 40] (Fig. 6g). However, again, there was not a significant pRab10 overlap with LAMP1 (Fig. 6h). Quantitation of colocalization of Rab10 with LAMP1 showed an MCC = 0.035, compared to pRab10 and LAMP1 (MCC = 0.0003, p = 0.004) (Fig. 6i). Rab10 and pRab10 did not colocalize with early endosome marker, EEA1 (Fig. 6j, k) with MCC = 0.0003 for Rab10/EEA1, and MCC = 0.0002 for pRab10/EEA1. There was no significant difference between MCC values (p = 0.4) (Fig. 6l).

### Rab10 and pRab10 at the presynaptic terminal

**Fig. 7:**
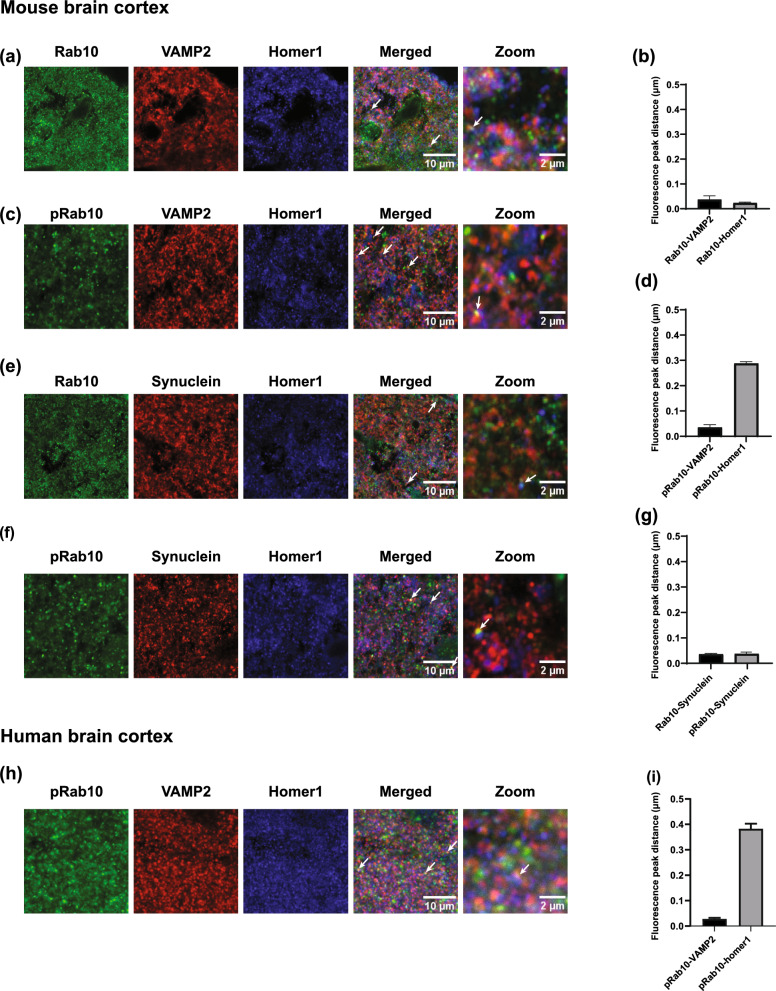
Immunofluorescence confocal image show Rab10 and pRab10 expression at the synapse in the C57BL/6J WT mouse brain. Triple labeling was performed (N = 3): Rab10/VAMP2/Homer or pRab10/VAMP2/Homer, with secondary antibodies anti-rabbit Alexa 488, anti-mouse Alexa 555, and anti-chicken Alexa 647. Here the Homer1 channel color was artificially changed to blue for all images. **a** Rab10 (green) colocalized with the presynaptic marker VAMP2 (red) and with the post synaptic marker Homer1 (blue), indicated by arrow, shown in merged and zoomed in image Scale bar = 10, 2 µm. **b** Distance between Rab10 and Vamp2 (0.0373 µm) and distance between Rab10 and Homer1 (0.0236 µm). **c** pRab10 (green) colocalized with the presynaptic marker VAMP2 (red), indicated by arrow but did not colocalize with the post synaptic marker Homer1 (blue), indicated by arrow, shown in merged and zoomed in image. Scale bar = 10, 2 µm. **d** Distance between pRab10 and Vamp2 (0.0353 µm) and between pRab10 and Homer1 (0.288 µm). **e** Rab10 (green) colocalized with α-synuclein (red) shown in merged and zoomed in image. Scale bar = 10, 2 µm. **f** pRab10 (green) colocalized with α synuclein (red) shown in merged and zoomed in image. Scale bar = 10, 2 µm. **g** Distance between Rab10 and α-synuclein (0.0361) and distance between pRab10 and α synuclein (0.0347 µm). (n = 3 biologically independent mouse brain samples). **h** In human brain temporal cortex, pRab10 (green) colocalized with the presynaptic marker VAMP2 (red), indicated by arrow but did not colocalized with the post synaptic marker Homer1 (blue), indicated by arrow shown in merged and zoomed in image. Scale bar = 10, 2 µm. **i** Distance between pRab10 and VAMP2 (0.0283 µm) and between pRab10 and Homer1 (0.383 µm). (n = 3 human brain samples)

LRRK2 localizes to and plays a role in synaptic vesicle trafficking [2, 10, 13, 49]. In addition, Rab10 has been shown to play a role in axonal anterograde traffic of vesicles to the axon tip [14]. To determine whether Rab10 and pRab10 localize to the presynaptic terminal or post-synaptic membrane, immunofluorescence was performed with VAMP2 and Homer1, known presynaptic and postsynaptic markers, respectively from three mouse brains (three sections per mouse). Because of difficulties resolving the pre-synaptic terminal from the post-synaptic density, measuring the co-localization with VAMP2 or Homer1 did not reveal any differences. We therefore determined the relative distance measurement between Rab10 and pRab10 and Vamp2 and Homer1 as previously using ImageJ plot profile tool [10]. To make sure the relative distance measurement was valid, a known presynaptic protein, α-synuclein, was used along with VAMP2 and Homer1. As expected, the relative distance measurement and quantitation shows that α-synuclein is more closely localized relative to VAMP2 (0.0467 µm distance) compared to Homer1 (0.346 µm distance) (Additional file 3). Immunofluorescence showed that Rab10 overlapped with both VAMP2 and Homer1 (Fig. 7a). To confirm the closeness of the Rab10 with VAMP2 and Homer1 a relative distance measurement was performed. The distance measurement quantitation between Rab10-VAMP2 and Rab10-Homer1, shows that Rab10 is in close proximity with both VAMP2 (0.0373 µm) and Homer1 (0.0236 µm) (Fig. 7b). Interestingly, pRab10 mainly localized with presynaptic marker VAMP2, but not with the postsynaptic marker Homer1 (Fig. 7c). Distance measurement and quantitation between pRab10-VAMP2 and pRab10-Homer1shows that pRab10 is in relatively closer proximity with VAMP2 (0.0353 µm) compared to Homer1 (0.288 µm) (Fig. 7d), suggesting pRab10 localizes more closely to the presynaptic membrane rather than post-synaptically. Similar quantitation was performed to find relative distance of Rab10 and pRab10 with α-synuclein, a presynaptic protein involved in PD. Immunofluorescence experiments and distance measurements show that both Rab10 and pRab10 localized with α-synuclein (Fig. 7 e,f). Quantitation of distance measurement indicates that Rab10 and pRab10 are in close proximity with α-synuclein as 0.0361 µm and 0.0347 µm respectively (Fig. 7g).

Finally, we examined pRab10 localization at the synapse in human brain temporal cortex. pRab10 showed overlap with presynaptic marker VAMP2 but not with Homer1, a postsynaptic marker (Fig. 7h). Distance measurement and quantitation of pRab10 with Vamp2 and Homer1 showed that pRab10 localized more closely VAMP2 (0.0283 µm) compared to Homer1 (0.383 µm) (Fig. 7i). Interestingly, pRab10 localization and its distance measurement at the synapse in the human brain samples were similar to the results from the mouse brain cortex.

## Discussion

Multiple studies show that a subset of Rab GTPases are LRRK2 kinase substrates, and LRRK2 can phosphorylate these Rab GTPases in vivo and in vitro [41, 79]. Rab10 specifically was identified as phosphorylated by LRRK2 combining two independent genetic and chemical phosphoproteomic screens and has been validated in subsequent studies [19, 28, 32, 34, 35, 41, 79]. Furthermore, a variant of Rab10 was identified as potentially protective in Alzheimer’s disease [64]. Because of the relatively low expression of Rab10, we utilized tyramide signal amplification to increase the signal and better visualize localization of pRab10. To assess specificity of the pRab10 antibody we performed Rab10 knock down experiments in the brain in vivo and utilized Rab10 KO iNs [47]. Our findings show that Rab10 and pRab10 are expressed in the cortex, striatum and SNc, brain regions involved in PD. Rab10 and pRab10 localize to excitatory neurons in the cortex, SPNs in the striatum, and dopaminergic neurons in the SNc as well as GABAergic and cholinergic interneurons. At the subcellular level, we showed Rab10 localized to the ER, TGN, and lysosomes, as expected, but pRab10 was not detectable in these organelles. pRab10, however, was detectable in a proportion of presynaptic terminals.

The absence of pRab10 signal at ER, TGN and lysosomes could result from the rapid action of PPMIH which dephosphorylates Rabs phosphorylated by LRRK2 [8]. At the presynaptic terminal however, our findings suggest that PPMIH activity is limited and that LRRK2-mediated phosphorylation of Rab10 is important for synaptic function. Multiple studies point to a role for LRRK2 at the presynaptic terminal where it plays a role in release of dopamine from striato-nigral neurons, and glutamate from corticostriatal neurons [5, 6, 11, 50, 86, 92, 95]. LRRK2 activity plays a role in synaptic vesicle endocytosis, activation of autophagy, and axonal transport of growth factor receptors [2, 7, 10, 24, 25, 36, 51, 59, 62, 75, 77, 96]. In the context of increased LRRK2 activity in PD, aberrations in these functions could lead to reduced availability of synaptic vesicles for neurotransmission, accumulation of damaged proteins and organelles and impaired transport of growth factor signaling to the cell body; all of which could result in neurodegeneration. While Rab3a, which plays a well-established role in synaptic vesicle endo-exocytosis is also phosphorylated by LRRK2, [16] our findings highlight a potentially important role for LRRK2-phosphorylated pRab10 at the presynaptic terminals.

Given the role of LRRK2 mutations in PD, in this study we focused on characterizing the localization of Rab10 and pRab10 in mouse brain areas primarily involved in PD pathology. We showed that Rab10 and pRab10 are expressed in striatum, cortex and SNc. The presence of Rab10 and pRab10 in the SNc and its enrichment in TH and DAT neurons indicate that it can play a role in these cells, which is important because loss of dopamine neurons is responsible for some of the motor symptoms in PD. In addition to the SNc, the striatum plays a major role in controlling movement. We found that Rab10 and pRab10 are both enriched in the spiny projection neurons of the striatum, consistent with a previous study from our group showing that LRRK2 is enriched in these neurons, particularly in the striosomes [48]. pRab10 and LRRK2 are also highly expressed in corticostriatal neurons and LRRK2 has been shown in multiple studies to play an important role in corticostriatal neurotransmission which is critical for basal ganglia function [11, 26, 48, 53, 54, 60, 63, 74, 90, 94]. Thus, future physiological studies on the role of LRRK2 and Rab10 in corticostriatal and SPN function will help understand the mechanisms by which aberrant LRRK2 activity contributes to PD.

Although interneurons only make up around 2–3% of the striatal cell population, they play an important role in modulating striatal transmission. We tested Rab10 and pRab10 prevalence in parvalbumin, calretinin, and acetylcholine expressing cells and found that they are present in both subtypes of interneurons. Abnormal LRRK2 kinase activity results in loss of primary cilia in these cholinergic interneurons [15, 30] and our data showing localization of pRab10 in these neurons helps support a role for pRab10 in suppressing ciliogenesis in response to hyperactive LRRK2 in cholinergic interneurons.

LRRK2 activity has been demonstrated in glial cells including astrocytes, microglia and oligodendrocytes [30, 55, 97], identified with markers GFAP, CD68 and Olig2. We found that Rab10 and pRab10 are detected in microglia, astrocytes and oligodendrocytes. In astrocytes, LRRK2 plays a role in ciliogenesis [15, 30, 35, 84]. Microglia, the resident immune cells in the brain, also play a role in clearance of α-synuclein, a process which is regulated by LRRK2 kinase activity [46, 71]. Recently, Rab10 has been shown to be involved in micropinocytosis, the process of internalizing small and soluble substances, that occurs in macrophages [44]. This process can reduce the pathological burden by clearing misfolded proteins including α-synuclein, tau and other cellular debris. Finally, Rab10 has been implicated in oligodendrocyte maturation [98] and LRRK2 phosphorylation of Rab10 may impact oligodendrocyte development and myelination in the central nervous system.

Studies show that phosphorylation not only interferes with the interaction of Rabs with its effector proteins, but in a few cases facilitates alternative interactions of Rabs with a completely new effector protein when they are phosphorylated [78]. For example, cargo adaptor proteins from the RILP (Rab interacting lysosomal protein like) family (RILPL1 and RILPL2) involved in ciliogenesis, bind tightly with some of the phosphorylated Rabs including Rab10, but not with the unphosphorylated Rabs [35, 78]. Another study shows that phosphorylation of Rab10 inhibits its interaction of EHBP1L1 and interferes with EHBP1L1 mediated recycling [44]. Moreover, LRRK2 phosphorylated Rab10 interacts with JIP3 and JIP4 (JNK interacting proteins), scaffold proteins for JNK signaling [89]. Given our results of enrichment of pRab10 at the presynaptic terminal, future studies to identify novel effectors that bind presynaptic Rab10 and pRab10 are highly needed.

## Conclusion

Leucine rich repeat kinase2 (LRRK2) mutations are one of the most common causes of familial PD, and abnormal LRRK2 has been implicated in idiopathic PD. Rab10 and its phosphorylated form, pRab10 likely play a role in neurodegenerative diseases including Parkinson’s and Alzheimer’s diseases [64, 79, 94]. In this study, we focused on characterizing Rab10 and pRab10 in the brain. We show that Rab10 and pRab10 are expressed in brain regions affected in PD such as SNc, striatum, and cortex. We also characterized Rab10 and pRab10 expression in different brain cell types like spiny projection neurons in the striatum, dopaminergic cells in SNc, excitatory neurons in the cortex, inhibitory neurons and glial cell types. Interestingly, pRab10 signal was only visible at the presynaptic terminal while Rab10 was visible at other organelles, suggesting differential LRRK2 and LRRK2 phosphatase activity in different cellular compartments. Our data open up avenues for research identifying novel presynaptic Rab10 effectors and determining how Rab10 and pRab10 affect functions including synaptic vesicle traffic, induction of autophagy and retrograde transport. Overall, the enrichment of pRab10 at the presynaptic terminal has potential implications for both PD and AD.

### Supplementary Information

**Additional file 1**. Intracerebroventricular injected C57Bl/6 WT mice with control ASO and Rab10 ASOs, ASO1 and ASO2, and G2019S-LRRK2 KI mice to confirm the specificity of the Rab10 and pRab10 antibodies. At 3–4 months of age, C57BL6/J WT mice received intraventricular injections with control and Rab10 specific ASOs. Mice were deeply anesthetized with vaporized isoflurane on a stereotactic frame. Mice were then injected with 10 µL of 30 µg/µL (total 300 µg) control and Rab10 ASOs using the coordinates + 0.3 mm AP, + 1.0 mm ML, -3.0 mm DV. Solutions were injected at a constant rate of 1 µL/min; once injection was complete, the needle was left in place for 5 min and then slowly withdrawn (**a**) Immunoblot of Rab10 in Rab10 ASOs (Rab10 ASO1 and Rab10 ASO2) and control ASO injected brain samples for cortex, midbrain and striatum. Tubulin was used as a loading control. (**b**) Quantitation of Rab10 protein and normalization with the loading control, tubulin, show reduction of the Rab10 in the Rab10 ASO1 compared to control ASO injected mouse samples in the cortex (*p* value = 0.0126), midbrain (*p* value = 0.0239) and in the striatum (*p* value = 0.0235) and reduction of the Rab10 in the Rab10 ASO2 compared to control ASO injected mouse samples in the cortex (*p* value = 0.0044), midbrain (*p* value = 0.0208) and in the striatum (*p* value = 0. 0133). One way ANOVA Dunnett’s multiple comparisons test was run for statistical analysis (n = 3 biologically independent samples). (**c**) Immunoblot of pRab10 in Rab10 ASOs (Rab10 ASO1 and Rab10 ASO2) and control ASO injected brain samples for cortex, midbrain and striatum. Tubulin was used as a loading control. (**d**) Quantitation of pRab10 protein and normalization with the loading control, tubulin, show reduction of the pRab10 in the Rab10 ASO1 compared to control ASO injected mouse samples in the cortex (*p* value = 0.0594), midbrain (*p* value = 0.0205) and in the striatum (*p* value = 0.1705) and reduction of the pRab10 in the Rab10 ASO2 compared to control ASO injected mouse samples in the cortex (*p* value = 0. 0544), midbrain (*p* value = 0.0192) and in the striatum (*p* value = 0.1087). One way ANOVA Dunnett’s multiple comparisons test was run for statistical analysis (n = 3 biologically independent samples). (**e**) Immunofluorescence for pRab10 (green) in the WT and G2019S KI mouse striatum region showed an increase of pRab10 in G2019S KI brain samples. NeuN (red) staining was used to show comparable immunofluorescence in the brain area. Scale bar = 500 µm. All images were captured at the same laser power, gain and offset. (**f**) The zoomed in image showed increased pRab10 (green) immunofluorescence in G2019S KI samples. Scale bar = 200 µm. All images were captured at the same laser power, gain and offset. (**g**) Quantitation of pRab10 integrated density immunofluorescence signal and normalization with NeuN signal showed a significant increase (*p* value = 0.004) in the G2019S KI samples (gray bar) compared to WT samples (black bar) (n = 3 biologically independent samples). For statistical analysis two tailed nested t-test was performed.

**Additional file 2**. Immunofluorescence and Immunoblot experiment. Immunofluorescence confocal images using 60 × oil objective show pRab10 antibody specificity in Rab10 KO iNs and in mouse brain. (**a**) pRab10 (green) colocalizes with the presynaptic marker VAMP2 (red) shown in merged and zoom image indicated by arrow in WT induced neurons. Scale bar 20, 5 µm. (**b**) pRab10 (green) staining with the pre-synaptic marker VAMP2 (red) shown in merged and zoom image in Rab10 KO induced neurons (n = 6 coverslips, 4 images were collected from each coverslip). (**c**) Mouse brain cortex area confocal image: Tyramide 488 staining (green) with pRab10 primary antibody, including anti Rabbit HRP conjugate and secondary antibodies, Alexa 555 goat anti mouse IgG, Alexa 647 goat anti chicken IgY. Zoom image from the tyramide 488 channel. (N = 3) (**d**) Mouse brain cortex area confocal image: Tyramide 488 staining (green) no primary antibody, including anti Rabbit HRP conjugate and secondary antibodies, Alexa 555 goat anti mouse IgG, Alexa 647 goat anti chicken IgY. Zoom image from the tyramide 488 channel. (N = 3) (**e**) Immunoblot for Rab8a and Rab10 in primary corticostriatal neurons treated with control ASO and Rab10 ASO-1. Hsc70 was used as a loading control. (**f**) Rab8a immunoblot signal was normalized with Hsc70 signal and quantitation plot shows control ASO in black bar and Rab10 ASO 1 in gray bar (N = 3, t-test, *p* value 0.56). Unpaired t-test with Welch’s correction was performed for the statistical analsysis. (g) Rab10 immunoblot signal was normalized with Hsc70 signal and quantitation plot shows control ASO in black bar and Rab10 ASO 1 in gray bar (N = 3, t-test, *p* value = 0.0001). Unpaired t-test with Welch’s correction was performed for the statistical analsysis.

**Additional file 3**. Immunofluorescence confocal images show α-synuclein expression at synapse in the C57Bl/6J WT mouse brain. (**a**) α-synuclein (green) colocalized with the presynaptic marker VAMP2 (red), indicated by arrow, but did not colocalize with the post synaptic marker Homer1 (blue), indicated by arrow, shown in merged and zoom image. Scale bar 10, 2 µm. (**c**) Distance between α-synuclein and VAMP2 (0.0467 µm) and distance between α-synuclein and Homer1 (0.346 µm). (n = 3 biologically independent samples).

**Additional file 4**. Movie showing the Rab10 in microglia cells (CD68 positive) in 3D view.

**Additional file 5**. Movie showing the pRab10 in microglia cells (CD68 positive) in 3D view.

**Additional file 6**. Movie showing the Rab10 in astrocyte (GFAP positive) in 3D view.

**Additional file 7**. Movie showing the pRab10 in astrocyte (GFAP positive) in 3D view.

## Data Availability

All primary data can be made available upon reasonable request.

## Abbreviations

AD: Alzheimer’s disease
ASO: Antisense oligo nucleotide
EEA1: Early endosome antigen1
ER: Endoplasmic reticulum
GAP: GTPase-activating proteins
GDI: GDP dissociation inhibitor
GEF: Guanine nucleotide exchange factors
INs: Induced neurons
LRRK2: Leucine rich repeat kinase 2
MCC: Mander’s colocalization coefficient
PD: Parkinson’s disease
SNc: Substantia Nigra pars compacta
SNr: Substantia Nigra pars reticulata
SPNs: Spiny projection neurons
TGN: Trans Golgi network
TSA: Tyramide signal amplification

## References

[1] Aosaki T, Miura M, Masuda M (2009). Physiological interaction between acetylcholine and dopamine in the striatum. Brain Nerve.

[2] Arranz AM, Delbroek L, Van Kolen K, Guimaraes MR, Mandemakers W, Daneels G, Matta S, Calafate S, Shaban H, Baatsen P (2015). LRRK2 functions in synaptic vesicle endocytosis through a kinase-dependent mechanism. J Cell Sci.

[3] Atashrazm F, Hammond D, Perera G, Bolliger MF, Matar E, Halliday GM, Schule B, Lewis SJG, Nichols RJ, Dzamko N (2019). LRRK2-mediated Rab10 phosphorylation in immune cells from Parkinson's disease patients. Mov Disord.

[4] Baba M, Nakajo S, Tu PH, Tomita T, Nakaya K, Lee VM, Trojanowski JQ, Iwatsubo T (1998). Aggregation of alpha-synuclein in Lewy bodies of sporadic Parkinson's disease and dementia with Lewy bodies. Am J Pathol.

[5] Beccano-Kelly DA, Kuhlmann N, Tatarnikov I, Volta M, Munsie LN, Chou P, Cao LP, Han H, Tapia L, Farrer MJ, Milnerwood AJ (2014). Synaptic function is modulated by LRRK2 and glutamate release is increased in cortical neurons of G2019S LRRK2 knock-in mice. Front Cell Neurosci.

[6] Beccano-Kelly DA, Volta M, Munsie LN, Paschall SA, Tatarnikov I, Co K, Chou P, Cao LP, Bergeron S, Mitchell E (2015). LRRK2 overexpression alters glutamatergic presynaptic plasticity, striatal dopamine tone, postsynaptic signal transduction, motor activity and memory. Hum Mol Genet.

[7] Belluzzi E, Gonnelli A, Cirnaru MD, Marte A, Plotegher N, Russo I, Civiero L, Cogo S, Carrion MP, Franchin C (2016). LRRK2 phosphorylates pre-synaptic N-ethylmaleimide sensitive fusion (NSF) protein enhancing its ATPase activity and SNARE complex disassembling rate. Mol Neurodegener.

[8] Berndsen K, Lis P, Yeshaw WM, Wawro PS, Nirujogi RS, Wightman M, Macartney T, Dorward M, Knebel A, Tonelli F (2019). PPM1H phosphatase counteracts LRRK2 signaling by selectively dephosphorylating Rab proteins. Elife.

[9] Bohnen NI, Albin RL (2011). The cholinergic system and Parkinson disease. Behav Brain Res.

[10] Brzozowski CF, Hijaz BA, Singh V, Gcwensa NZ, Kelly K, Boyden ES, West AB, Sarkar D, Volpicelli-Daley LA (2021). Inhibition of LRRK2 kinase activity promotes anterograde axonal transport and presynaptic targeting of alpha-synuclein. Acta Neuropathol Commun.

[11] Chen C, Soto G, Dumrongprechachan V, Bannon N, Kang S, Kozorovitskiy Y, Parisiadou L (2020). Pathway-specific dysregulation of striatal excitatory synapses by LRRK2 mutations. Elife.

[12] Chen CC, Schweinsberg PJ, Vashist S, Mareiniss DP, Lambie EJ, Grant BD (2006). RAB-10 is required for endocytic recycling in the Caenorhabditis elegans intestine. Mol Biol Cell.

[13] Cirnaru MD, Marte A, Belluzzi E, Russo I, Gabrielli M, Longo F, Arcuri L, Murru L, Bubacco L, Matteoli M (2014). LRRK2 kinase activity regulates synaptic vesicle trafficking and neurotransmitter release through modulation of LRRK2 macro-molecular complex. Front Mol Neurosci.

[14] Deng CY, Lei WL, Xu XH, Ju XC, Liu Y, Luo ZG (2014). JIP1 mediates anterograde transport of Rab10 cargos during neuronal polarization. J Neurosci.

[15] Dhekne HS, Yanatori I, Gomez RC, Tonelli F, Diez F, Schule B, Steger M, Alessi DR, Pfeffer SR (2018). A pathway for Parkinson's Disease LRRK2 kinase to block primary cilia and Sonic hedgehog signaling in the brain. Elife.

[16] Dou D, Aiken J, Holzbaur ELF (2023) RAB3 phosphorylation by pathogenic LRRK2 impairs trafficking of synaptic vesicle precursors. bioRxiv. 10.1101/2023.07.25.550521 10.1083/jcb.202307092 PMC10959120 38512027

[17] English AR, Voeltz GK (2013). Rab10 GTPase regulates ER dynamics and morphology. Nat Cell Biol.

[18] Etoh K, Fukuda M (2019). Rab10 regulates tubular endosome formation through KIF13A and KIF13B motors. J Cell Sci.

[19] Fan Y, Howden AJM, Sarhan AR, Lis P, Ito G, Martinez TN, Brockmann K, Gasser T, Alessi DR, Sammler EM (2018). Interrogating Parkinson's disease LRRK2 kinase pathway activity by assessing Rab10 phosphorylation in human neutrophils. Biochem J.

[20] Fan Y, Nirujogi RS, Garrido A, Ruiz-Martinez J, Bergareche-Yarza A, Mondragon-Rezola E, Vinagre-Aragon A, Croitoru I, Gorostidi Pagola A, Paternain Markinez L (2021). R1441G but not G2019S mutation enhances LRRK2 mediated Rab10 phosphorylation in human peripheral blood neutrophils. Acta Neuropathol.

[21] Glodowski DR, Chen CC, Schaefer H, Grant BD, Rongo C (2007). RAB-10 regulates glutamate receptor recycling in a cholesterol-dependent endocytosis pathway. Mol Biol Cell.

[22] Gustafson EL, Ehrlich ME, Trivedi P, Greengard P (1992). Developmental regulation of phosphoprotein gene expression in the caudate-putamen of rat: an in situ hybridization study. Neuroscience.

[23] Healy DG, Falchi M, O'Sullivan SS, Bonifati V, Durr A, Bressman S, Brice A, Aasly J, Zabetian CP, Goldwurm S (2008). Phenotype, genotype, and worldwide genetic penetrance of LRRK2-associated Parkinson's disease: a case-control study. Lancet Neurol.

[24] Heaton GR, Landeck N, Mamais A, Nalls MA, Nixon-Abell J, Kumaran R, Beilina A, Pellegrini L, Li Y, International Parkinson Disease Genomics Cet al (2020) Sequential screening nominates the Parkinson's disease associated kinase LRRK2 as a regulator of Clathrin-mediated endocytosis. Neurobiol Dis 141:104948. 10.1016/j.nbd.2020.104948 10.1016/j.nbd.2020.104948 PMC7339134 32434048

[25] Hernandez-Diaz S, Ghimire S, Sanchez-Mirasierra I, Montecinos-Oliva C, Swerts J, Kuenen S, Verstreken P, Soukup SF (2022). Endophilin-B regulates autophagy during synapse development and neurodegeneration. Neurobiol Dis.

[26] Higashi S, Moore DJ, Colebrooke RE, Biskup S, Dawson VL, Arai H, Dawson TM, Emson PC (2007). Expression and localization of Parkinson's disease-associated leucine-rich repeat kinase 2 in the mouse brain. J Neurochem.

[27] Huang Y, Song NN, Lan W, Hu L, Su CJ, Ding YQ, Zhang L (2013). Expression of transcription factor Satb2 in adult mouse brain. Anat Rec (Hoboken).

[28] Ito G, Katsemonova K, Tonelli F, Lis P, Baptista MA, Shpiro N, Duddy G, Wilson S, Ho PW, Ho SL (2016). Phos-tag analysis of Rab10 phosphorylation by LRRK2: a powerful assay for assessing kinase function and inhibitors. Biochem J.

[29] Karunanithi S, Xiong T, Uhm M, Leto D, Sun J, Chen XW, Saltiel AR (2014). A Rab10:RalA G protein cascade regulates insulin-stimulated glucose uptake in adipocytes. Mol Biol Cell.

[30] Khan SS, Sobu Y, Dhekne HS, Tonelli F, Berndsen K, Alessi DR, Pfeffer SR (2021). Pathogenic LRRK2 control of primary cilia and Hedgehog signaling in neurons and astrocytes of mouse brain. Elife.

[31] Kumari U, Tan EK (2009). LRRK2 in Parkinson's disease: genetic and clinical studies from patients. FEBS J.

[32] Kuwahara T, Funakawa K, Komori T, Sakurai M, Yoshii G, Eguchi T, Fukuda M, Iwatsubo T (2020). Roles of lysosomotropic agents on LRRK2 activation and Rab10 phosphorylation. Neurobiol Dis.

[33] Lagomarsino VN, Pearse RV, Liu L, Hsieh YC, Fernandez MA, Vinton EA, Paull D, Felsky D, Tasaki S, Gaiteri C (2021). Stem cell-derived neurons reflect features of protein networks, neuropathology, and cognitive outcome of their aged human donors. Neuron.

[34] Lara Ordonez AJ, Fasiczka R, Fernandez B, Naaldijk Y, Fdez E, Blanca Ramirez M, Phan S, Boassa D, Hilfiker S (2022). The LRRK2 signaling network converges on a centriolar phospho-Rab10/RILPL1 complex to cause deficits in centrosome cohesion and cell polarization. Biol Open.

[35] Lara Ordonez AJ, Fernandez B, Fdez E, Romo-Lozano M, Madero-Perez J, Lobbestael E, Baekelandt V, Aiastui A, Lopez de Munain A, Melrose HL (2019). RAB8, RAB10 and RILPL1 contribute to both LRRK2 kinase-mediated centrosomal cohesion and ciliogenesis deficits. Hum Mol Genet.

[36] Lazo OM, Schiavo G (2023). Rab10 regulates the sorting of internalised TrkB for retrograde axonal transport. Elife.

[37] Lee H, Flynn R, Sharma I, Haberman E, Carling PJ, Nicholls FJ, Stegmann M, Vowles J, Haenseler W, Wade-Martins R (2020). LRRK2 Is Recruited to Phagosomes and Co-recruits RAB8 and RAB10 in Human Pluripotent Stem Cell-Derived Macrophages. Stem Cell Rep.

[38] Lesage S, Durr A, Tazir M, Lohmann E, Leutenegger AL, Janin S, Pollak P, Brice A, French Parkinson's Disease Genetics Study G (2006). LRRK2 G2019S as a cause of Parkinson's disease in North African Arabs. N Engl J Med.

[39] Lesage S, Ibanez P, Lohmann E, Pollak P, Tison F, Tazir M, Leutenegger AL, Guimaraes J, Bonnet AM, Agid Y (2005). G2019S LRRK2 mutation in French and North African families with Parkinson's disease. Ann Neurol.

[40] Li Z, Schulze RJ, Weller SG, Krueger EW, Schott MB, Zhang X, Casey CA, Liu J, Stockli J, James DE, McNiven MA (2016). A novel Rab10-EHBP1-EHD2 complex essential for the autophagic engulfment of lipid droplets. Sci Adv.

[41] Lis P, Burel S, Steger M, Mann M, Brown F, Diez F, Tonelli F, Holton JL, Ho PW, Ho SL (2018). Development of phospho-specific Rab protein antibodies to monitor in vivo activity of the LRRK2 Parkinson's disease kinase. Biochem J.

[42] Liu O, Grant BD (2015). Basolateral Endocytic Recycling Requires RAB-10 and AMPH-1 Mediated Recruitment of RAB-5 GAP TBC-2 to Endosomes. PLoS Genet.

[43] Liu Y, Xu XH, Chen Q, Wang T, Deng CY, Song BL, Du JL, Luo ZG (2013). Myosin Vb controls biogenesis of post-Golgi Rab10 carriers during axon development. Nat Commun.

[44] Liu Z, Xu E, Zhao HT, Cole T, West AB (2020). LRRK2 and Rab10 coordinate macropinocytosis to mediate immunological responses in phagocytes. EMBO J.

[45] Lv P, Sheng Y, Zhao Z, Zhao W, Gu L, Xu T, Song E (2015). Targeted disruption of Rab10 causes early embryonic lethality. Protein Cell.

[46] Maekawa T, Sasaoka T, Azuma S, Ichikawa T, Melrose HL, Farrer MJ, Obata F (2016). Leucine-rich repeat kinase 2 (LRRK2) regulates alpha-synuclein clearance in microglia. BMC Neurosci.

[47] Mamais A, Sanyal A, Fajfer A, Zykoski CG, Guldin M, Riley-DiPaolo A, Subrahmanian N, Gibbs W, Lin S, LaVoie MJ (2023) The LRRK2 kinase substrates Rab8a and Rab10 contribute complementary but distinct disease-relevant phenotypes in human neurons. bioRxiv. 10.1101/2023.04.30.538317 10.1016/j.stemcr.2024.01.001 PMC10874859 38307024

[48] Mandemakers W, Snellinx A, O'Neill MJ, de Strooper B (2012). LRRK2 expression is enriched in the striosomal compartment of mouse striatum. Neurobiol Dis.

[49] Matikainen-Ankney BA, Kezunovic N, Menard C, Flanigan ME, Zhong Y, Russo SJ, Benson DL, Huntley GW (2018). Parkinson's disease-linked LRRK2-G2019S mutation alters synaptic plasticity and promotes resilience to chronic social stress in young adulthood. J Neurosci.

[50] Matikainen-Ankney BA, Kezunovic N, Mesias RE, Tian Y, Williams FM, Huntley GW, Benson DL (2016). Altered development of synapse structure and function in striatum caused by Parkinson's disease-linked LRRK2-G2019S mutation. J Neurosci.

[51] Matta S, Van Kolen K, da Cunha R, van den Bogaart G, Mandemakers W, Miskiewicz K, De Bock PJ, Morais VA, Vilain S, Haddad D (2012). LRRK2 controls an EndoA phosphorylation cycle in synaptic endocytosis. Neuron.

[52] McRitchie DA, Cartwright HR, Halliday GM (1997). Specific A10 dopaminergic nuclei in the midbrain degenerate in Parkinson's disease. Exp Neurol.

[53] Melrose HL, Dachsel JC, Behrouz B, Lincoln SJ, Yue M, Hinkle KM, Kent CB, Korvatska E, Taylor JP, Witten L (2010). Impaired dopaminergic neurotransmission and microtubule-associated protein tau alterations in human LRRK2 transgenic mice. Neurobiol Dis.

[54] Migheli R, Del Giudice MG, Spissu Y, Sanna G, Xiong Y, Dawson TM, Dawson VL, Galioto M, Rocchitta G, Biosa A (2013). LRRK2 affects vesicle trafficking, neurotransmitter extracellular level and membrane receptor localization. PLoS ONE.

[55] Moehle MS, Webber PJ, Tse T, Sukar N, Standaert DG, DeSilva TM, Cowell RM, West AB (2012). LRRK2 inhibition attenuates microglial inflammatory responses. J Neurosci.

[56] Ouimet CC, Langley-Gullion KC, Greengard P (1998). Quantitative immunocytochemistry of DARPP-32-expressing neurons in the rat caudatoputamen. Brain Res.

[57] Ozelius LJ, Senthil G, Saunders-Pullman R, Ohmann E, Deligtisch A, Tagliati M, Hunt AL, Klein C, Henick B, Hailpern SM (2006). LRRK2 G2019S as a cause of Parkinson's disease in Ashkenazi Jews. N Engl J Med.

[58] Paisan-Ruiz C, Lewis PA, Singleton AB (2013). LRRK2: cause, risk, and mechanism. J Parkinsons Dis.

[59] Pan PY, Li X, Wang J, Powell J, Wang Q, Zhang Y, Chen Z, Wicinski B, Hof P, Ryan TA, Yue Z (2017). Parkinson's Disease-Associated LRRK2 Hyperactive Kinase Mutant Disrupts Synaptic Vesicle Trafficking in Ventral Midbrain Neurons. J Neurosci.

[60] Parisiadou L, Yu J, Sgobio C, Xie C, Liu G, Sun L, Gu XL, Lin X, Crowley NA, Lovinger DM, Cai H (2014). LRRK2 regulates synaptogenesis and dopamine receptor activation through modulation of PKA activity. Nat Neurosci.

[61] Phelps PE, Houser CR, Vaughn JE (1985). Immunocytochemical localization of choline acetyltransferase within the rat neostriatum: a correlated light and electron microscopic study of cholinergic neurons and synapses. J Comp Neurol.

[62] Piccoli G, Onofri F, Cirnaru MD, Kaiser CJ, Jagtap P, Kastenmuller A, Pischedda F, Marte A, von Zweydorf F, Vogt A (2014). Leucine-rich repeat kinase 2 binds to neuronal vesicles through protein interactions mediated by its C-terminal WD40 domain. Mol Cell Biol.

[63] Rassu M, Del Giudice MG, Sanna S, Taymans JM, Morari M, Brugnoli A, Frassineti M, Masala A, Esposito S, Galioto M (2017). Role of LRRK2 in the regulation of dopamine receptor trafficking. PLoS ONE.

[64] Ridge PG, Karch CM, Hsu S, Arano I, Teerlink CC, Ebbert MTW, Gonzalez Murcia JD, Farnham JM, Damato AR, Allen M (2017). Linkage, whole genome sequence, and biological data implicate variants in RAB10 in Alzheimer's disease resilience. Genome Med.

[65] Rizzi G, Tan KR (2017). Dopamine and Acetylcholine, a Circuit Point of View in Parkinson's Disease. Front Neural Circuits.

[66] Rymar VV, Sasseville R, Luk KC, Sadikot AF (2004). Neurogenesis and stereological morphometry of calretinin-immunoreactive GABAergic interneurons of the neostriatum. J Comp Neurol.

[67] Sano H, Eguez L, Teruel MN, Fukuda M, Chuang TD, Chavez JA, Lienhard GE, McGraw TE (2007). Rab10, a target of the AS160 Rab GAP, is required for insulin-stimulated translocation of GLUT4 to the adipocyte plasma membrane. Cell Metab.

[68] Santpere G, Ferrer I (2009). LRRK2 and neurodegeneration. Acta Neuropathol.

[69] Sanyal A, Novis HS, Gasser E, Lin S, LaVoie MJ (2020). LRRK2 kinase inhibition rescues deficits in lysosome function due to heterozygous GBA1 expression in human iPSC-derived neurons. Front Neurosci.

[70] Sato T, Iwano T, Kunii M, Matsuda S, Mizuguchi R, Jung Y, Hagiwara H, Yoshihara Y, Yuzaki M, Harada R, Harada A (2014). Rab8a and Rab8b are essential for several apical transport pathways but insufficient for ciliogenesis. J Cell Sci.

[71] Schapansky J, Nardozzi JD, LaVoie MJ (2015). The complex relationships between microglia, alpha-synuclein, and LRRK2 in Parkinson's disease. Neuroscience.

[72] Schuck S, Gerl MJ, Ang A, Manninen A, Keller P, Mellman I, Simons K (2007). Rab10 is involved in basolateral transport in polarized Madin-Darby canine kidney cells. Traffic.

[73] Simon-Sanchez J, Herranz-Perez V, Olucha-Bordonau F, Perez-Tur J (2006). LRRK2 is expressed in areas affected by Parkinson's disease in the adult mouse brain. Eur J Neurosci.

[74] Skelton PD, Tokars V, Parisiadou L (2022). LRRK2 at striatal synapses: cell-type specificity and mechanistic insights. Cells.

[75] Soukup SF, Kuenen S, Vanhauwaert R, Manetsberger J, Hernandez-Diaz S, Swerts J, Schoovaerts N, Vilain S, Gounko NV, Vints K (2016). A LRRK2-dependent endophilina phosphoswitch is critical for macroautophagy at presynaptic terminals. Neuron.

[76] Spillantini MG, Schmidt ML, Lee VM, Trojanowski JQ, Jakes R, Goedert M (1997). Alpha-synuclein in Lewy bodies. Nature.

[77] Stafa K, Tsika E, Moser R, Musso A, Glauser L, Jones A, Biskup S, Xiong Y, Bandopadhyay R, Dawson VL (2014). Functional interaction of Parkinson's disease-associated LRRK2 with members of the dynamin GTPase superfamily. Hum Mol Genet.

[78] Steger M, Diez F, Dhekne HS, Lis P, Nirujogi RS, Karayel O, Tonelli F, Martinez TN, Lorentzen E, Pfeffer SR (2017). Systematic proteomic analysis of LRRK2-mediated Rab GTPase phosphorylation establishes a connection to ciliogenesis. Elife.

[79] Steger M, Tonelli F, Ito G, Davies P, Trost M, Vetter M, Wachter S, Lorentzen E, Duddy G, Wilson S (2016). Phosphoproteomics reveals that Parkinson's disease kinase LRRK2 regulates a subset of Rab GTPases. Elife.

[80] Streubel-Gallasch L, Giusti V, Sandre M, Tessari I, Plotegher N, Giusto E, Masato A, Iovino L, Battisti I, Arrigoni G (2021). Parkinson's disease-associated LRRK2 interferes with astrocyte-mediated alpha-synuclein clearance. Mol Neurobiol.

[81] Swayze EE, Siwkowski AM, Wancewicz EV, Migawa MT, Wyrzykiewicz TK, Hung G, Monia BP, Bennett CF (2007). Antisense oligonucleotides containing locked nucleic acid improve potency but cause significant hepatotoxicity in animals. Nucl Acids Res.

[82] Szalisznyo K, Muller L (2009). Dopamine induced switch in the subthreshold dynamics of the striatal cholinergic interneurons: a numerical study. J Theor Biol.

[83] Tavana JP, Rosene M, Jensen NO, Ridge PG, Kauwe JS, Karch CM (2019). RAB10: an Alzheimer's disease resilience locus and potential drug target. Clin Interv Aging.

[84] Tremblay ME, Cookson MR, Civiero L (2019). Glial phagocytic clearance in Parkinson's disease. Mol Neurodegener.

[85] Volpicelli-Daley LA, Luk KC, Lee VM (2014). Addition of exogenous alpha-synuclein preformed fibrils to primary neuronal cultures to seed recruitment of endogenous alpha-synuclein to Lewy body and Lewy neurite-like aggregates. Nat Protoc.

[86] Volta M, Beccano-Kelly DA, Paschall SA, Cataldi S, MacIsaac SE, Kuhlmann N, Kadgien CA, Tatarnikov I, Fox J, Khinda J (2017). Initial elevations in glutamate and dopamine neurotransmission decline with age, as does exploratory behavior, in LRRK2 G2019S knock-in mice. Elife.

[87] Wang D, Lou J, Ouyang C, Chen W, Liu Y, Liu X, Cao X, Wang J, Lu L (2010). Ras-related protein Rab10 facilitates TLR4 signaling by promoting replenishment of TLR4 onto the plasma membrane. Proc Natl Acad Sci USA.

[88] Wang T, Liu Y, Xu XH, Deng CY, Wu KY, Zhu J, Fu XQ, He M, Luo ZG (2011). Lgl1 activation of rab10 promotes axonal membrane trafficking underlying neuronal polarization. Dev Cell.

[89] Waschbusch D, Purlyte E, Pal P, McGrath E, Alessi DR, Khan AR (2020). Structural Basis for Rab8a Recruitment of RILPL2 via LRRK2 Phosphorylation of Switch 2. Structure.

[90] West AB, Cowell RM, Daher JP, Moehle MS, Hinkle KM, Melrose HL, Standaert DG, Volpicelli-Daley LA (2014). Differential LRRK2 expression in the cortex, striatum, and substantia nigra in transgenic and nontransgenic rodents. J Comp Neurol.

[91] West AB, Moore DJ, Biskup S, Bugayenko A, Smith WW, Ross CA, Dawson VL, Dawson TM (2005). Parkinson's disease-associated mutations in leucine-rich repeat kinase 2 augment kinase activity. Proc Natl Acad Sci USA.

[92] Xenias HS, Chen C, Kang S, Cherian S, Situ X, Shanmugasundaram B, Liu G, Scesa G, Savio Chan C, Parisiadou L (2022). R1441C and G2019S LRRK2 knockin mice have distinct striatal molecular, physiological, and behavioral alterations. Commun Biol.

[93] Xu XH, Deng CY, Liu Y, He M, Peng J, Wang T, Yuan L, Zheng ZS, Blackshear PJ, Luo ZG (2014). MARCKS regulates membrane targeting of Rab10 vesicles to promote axon development. Cell Res.

[94] Yan T, Wang L, Gao J, Siedlak SL, Huntley ML, Termsarasab P, Perry G, Chen SG, Wang X (2018). Rab10 phosphorylation is a prominent pathological feature in Alzheimer's disease. J Alzheimers Dis.

[95] Yue M, Hinkle KM, Davies P, Trushina E, Fiesel FC, Christenson TA, Schroeder AS, Zhang L, Bowles E, Behrouz B (2015). Progressive dopaminergic alterations and mitochondrial abnormalities in LRRK2 G2019S knock-in mice. Neurobiol Dis.

[96] Yun HJ, Park J, Ho DH, Kim H, Kim CH, Oh H, Ga I, Seo H, Chang S, Son I, Seol W (2013). LRRK2 phosphorylates Snapin and inhibits interaction of Snapin with SNAP-25. Exp Mol Med.

[97] Zaldivar-Diez J, Li L, Garcia AM, Zhao WN, Medina-Menendez C, Haggarty SJ, Gil C, Morales AV, Martinez A (2020). Benzothiazole-based LRRK2 inhibitors as wnt enhancers and promoters of oligodendrocytic fate. J Med Chem.

[98] Zhang ZH, Zhao WQ, Ma FF, Zhang H, Xu XH (2017). Rab10 Disruption Results in Delayed OPC Maturation. Cell Mol Neurobiol.

[99] Zimprich A, Biskup S, Leitner P, Lichtner P, Farrer M, Lincoln S, Kachergus J, Hulihan M, Uitti RJ, Calne DB (2004). Mutations in LRRK2 cause autosomal-dominant parkinsonism with pleomorphic pathology. Neuron.

