# Supplementary figures and images for: Cellular and subcellular localization of Rab10 and phospho-T73 Rab10 in the mouse and human brain
Source: Acta Neuropathol Commun. 2023 Dec 18;11:201. doi: 10.1186/s40478-023-01704-9 (PMC10726543; doi:10.1186/s40478-023-01704-9)

Additional file 1

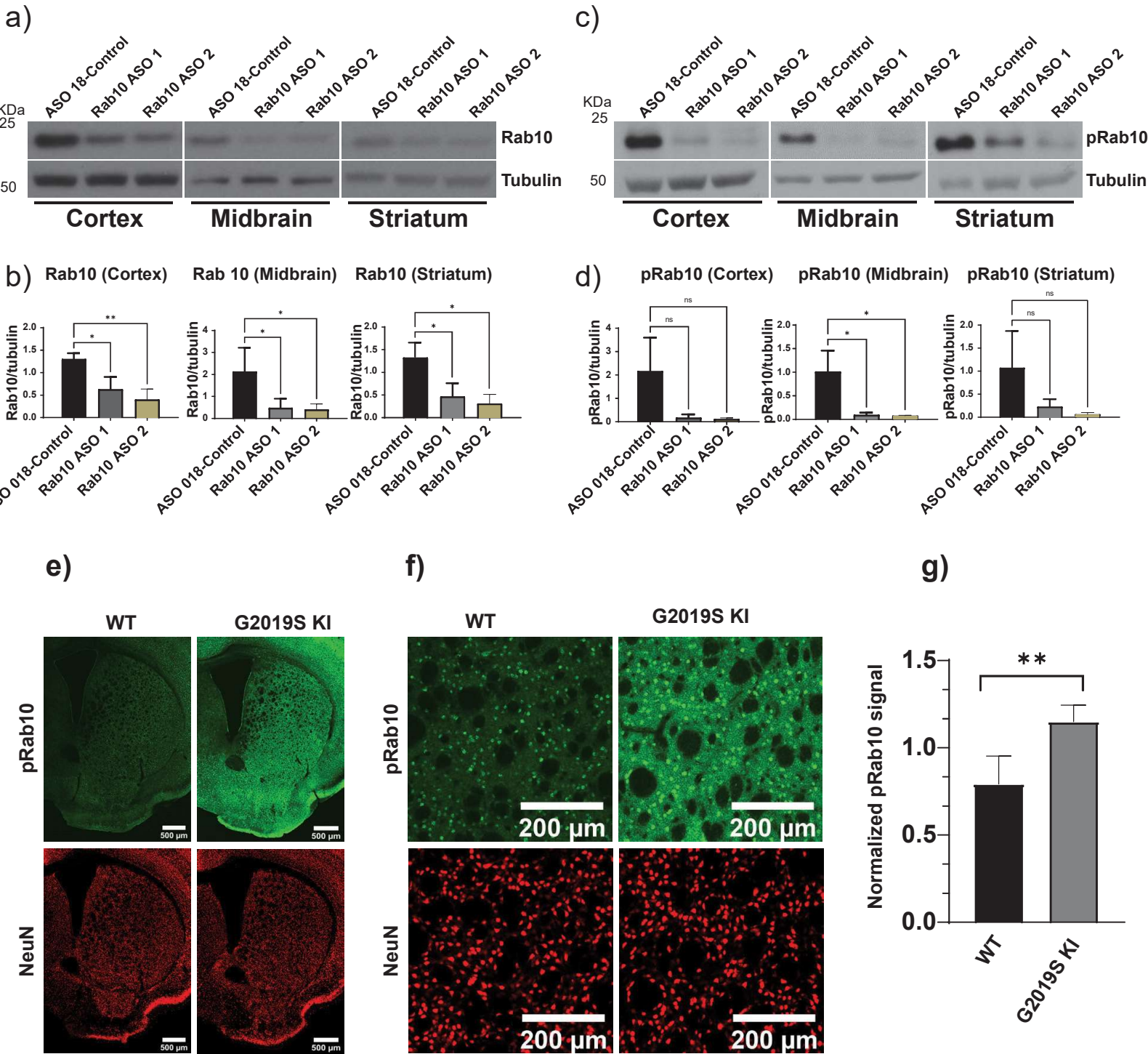

Supplement: Supplementary file 1 — Additional file 1. Intracerebroventricular injected C57Bl/6 WT mice with control ASO and Rab10 ASOs, ASO1 and ASO2, and G2019S-LRRK2 KI mice to confirm the specificity of the Rab10 and pRab10 antibodies. At 3–4 months of age, C57BL6/J WT mice received intraventricular injections with control and Rab10 specific ASOs. Mice were deeply anesthetized with vaporized isoflurane on a stereotactic frame. Mice were then injected with 10 µL of 30 µg/µL (total 300 µg) control and Rab10 ASOs using the coordinates + 0.3 mm AP, + 1.0 mm ML, -3.0 mm DV. Solutions were injected at a constant rate of 1 µL/min; once injection was complete, the needle was left in place for 5 min and then slowly withdrawn (a) Immunoblot of Rab10 in Rab10 ASOs (Rab10 ASO1 and Rab10 ASO2) and control ASO injected brain samples for cortex, midbrain and striatum. Tubulin was used as a loading control. (b) Quantitation of Rab10 protein and normalization with the loading control, tubulin, show reduction of the Rab10 in the Rab10 ASO1 compared to control ASO injected mouse samples in the cortex (p value = 0.0126), midbrain (p value = 0.0239) and in the striatum (p value = 0.0235) and reduction of the Rab10 in the Rab10 ASO2 compared to control ASO injected mouse samples in the cortex (p value = 0.0044), midbrain (p value = 0.0208) and in the striatum (p value = 0. 0133). One way ANOVA Dunnett’s multiple comparisons test was run for statistical analysis (n = 3 biologically independent samples). (c) Immunoblot of pRab10 in Rab10 ASOs (Rab10 ASO1 and Rab10 ASO2) and control ASO injected brain samples for cortex, midbrain and striatum. Tubulin was used as a loading control. (d) Quantitation of pRab10 protein and normalization with the loading control, tubulin, show reduction of the pRab10 in the Rab10 ASO1 compared to control ASO injected mouse samples in the cortex (p value = 0.0594), midbrain (p value = 0.0205) and in the striatum (p value = 0.1705) and reduction of the pRab10 in the Rab10 ASO2 compared to contr [file 40478_2023_1704_MOESM1_ESM.pdf]

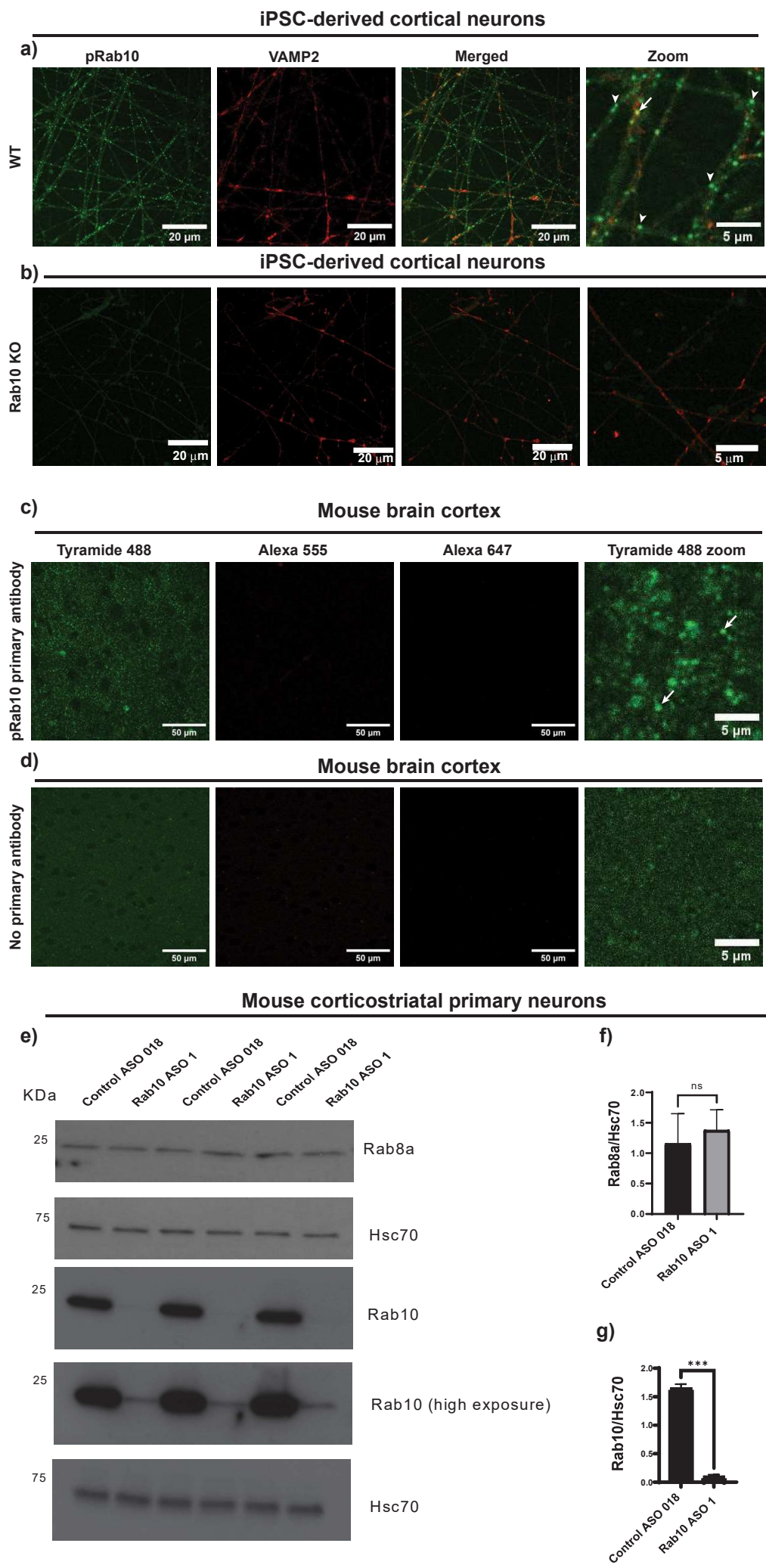

Supplement: Supplementary file 2 — Additional file 2. Immunofluorescence and Immunoblot experiment. Immunofluorescence confocal images using 60 × oil objective show pRab10 antibody specificity in Rab10 KO iNs and in mouse brain. (a) pRab10 (green) colocalizes with the presynaptic marker VAMP2 (red) shown in merged and zoom image indicated by arrow in WT induced neurons. Scale bar 20, 5 µm. (b) pRab10 (green) staining with the pre-synaptic marker VAMP2 (red) shown in merged and zoom image in Rab10 KO induced neurons (n = 6 coverslips, 4 images were collected from each coverslip). (c) Mouse brain cortex area confocal image: Tyramide 488 staining (green) with pRab10 primary antibody, including anti Rabbit HRP conjugate and secondary antibodies, Alexa 555 goat anti mouse IgG, Alexa 647 goat anti chicken IgY. Zoom image from the tyramide 488 channel. (N = 3) (d) Mouse brain cortex area confocal image: Tyramide 488 staining (green) no primary antibody, including anti Rabbit HRP conjugate and secondary antibodies, Alexa 555 goat anti mouse IgG, Alexa 647 goat anti chicken IgY. Zoom image from the tyramide 488 channel. (N = 3) (e) Immunoblot for Rab8a and Rab10 in primary corticostriatal neurons treated with control ASO and Rab10 ASO-1. Hsc70 was used as a loading control. (f) Rab8a immunoblot signal was normalized with Hsc70 signal and quantitation plot shows control ASO in black bar and Rab10 ASO 1 in gray bar (N = 3, t-test, p value 0.56). Unpaired t-test with Welch’s correction was performed for the statistical analsysis. (g) Rab10 immunoblot signal was normalized with Hsc70 signal and quantitation plot shows control ASO in black bar and Rab10 ASO 1 in gray bar (N = 3, t-test, p value = 0.0001). Unpaired t-test with Welch’s correction was performed for the statistical analsysis. [file 40478_2023_1704_MOESM2_ESM.pdf]

Additional file 3

a

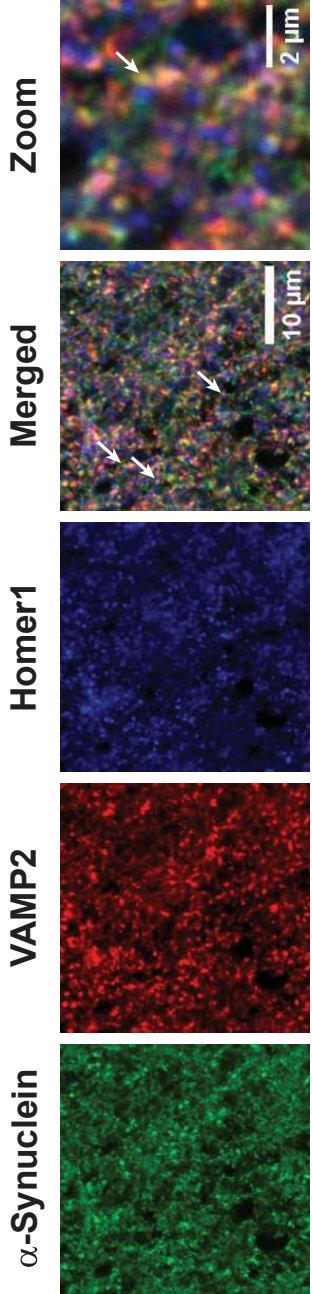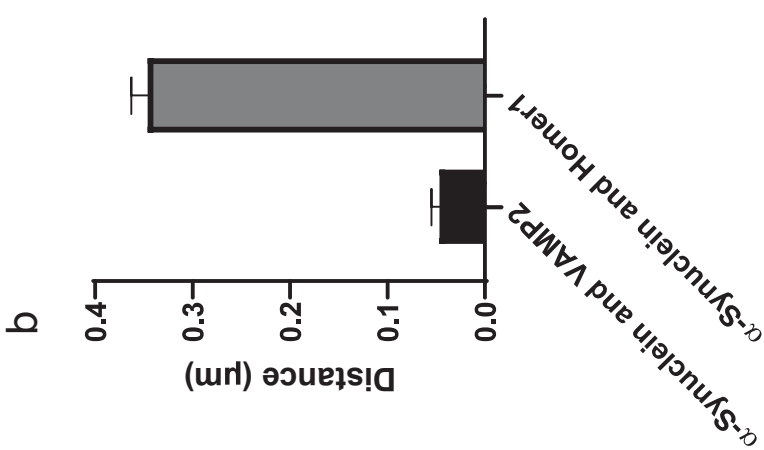

Supplement: Supplementary file 3 — Additional file 3. Immunofluorescence confocal images show α-synuclein expression at synapse in the C57Bl/6J WT mouse brain. (a) α-synuclein (green) colocalized with the presynaptic marker VAMP2 (red), indicated by arrow, but did not colocalize with the post synaptic marker Homer1 (blue), indicated by arrow, shown in merged and zoom image. Scale bar 10, 2 µm. (c) Distance between α-synuclein and VAMP2 (0.0467 µm) and distance between α-synuclein and Homer1 (0.346 µm). (n = 3 biologically independent samples). [file 40478_2023_1704_MOESM3_ESM.pdf]
